# MapB, the *Brucella suis* TamB homologue, is involved in cell envelope biogenesis, cell division and virulence

**DOI:** 10.1038/s41598-018-37668-3

**Published:** 2019-02-15

**Authors:** Magalí Graciela Bialer, Verónica Ruiz-Ranwez, Gabriela Sycz, Silvia Marcela Estein, Daniela Marta Russo, Silvia Altabe, Rodrigo Sieira, Angeles Zorreguieta

**Affiliations:** 1 Fundación Instituto Leloir, IIBBA-CONICET. Patricias Argentinas 435, (C1405BWE), Buenos Aires, Argentina; 2 Laboratorio de Inmunología, Facultad de Ciencias Veterinarias, Centro de Investigación Veterinaria de Tandil (CIVETAN), CONICET-Facultad de Ciencias Veterinarias, Universidad Nacional del Centro de la Provincia de Buenos Aires (U.N.C.P.B.A), Tandil, Argentina; 3 Instituto de Biología Molecular y Celular de Rosario (IBR) and Departamento de Microbiología, Facultad de Ciencias Bioquímicas y Farmacéuticas, Universidad Nacional de Rosario, Esmeralda y Ocampo, Rosario, Argentina; 4 Departamento de Química Biológica, Facultad de Ciencias Exactas y Naturales, Universidad de Buenos Aires, Buenos Aires, Argentina

## Abstract

*Brucella* species are Gram-negative, facultative intracellular pathogens responsible for a worldwide zoonosis. The envelope of *Brucella* exhibits unique characteristics that make these bacteria furtive pathogens and resistant to several host defence compounds. We have identified a *Brucella suis* gene (*mapB*) that appeared to be crucial for cell envelope integrity. Indeed, the typical resistance of *Brucella* to both lysozyme and the cationic lipopeptide polymyxin B was markedly reduced in a ∆*mapB* mutant. MapB turned out to represent a TamB orthologue. This last protein, together with TamA, a protein belonging to the Omp85 family, form a complex that has been proposed to participate in the translocation of autotransporter proteins across the outer membrane (OM). Accordingly, we observed that MapB is required for proper assembly of an autotransporter adhesin in the OM, as most of the autotransporter accumulated in the mutant cell periplasm. Both assessment of the relative amounts of other specific outer membrane proteins (OMPs) and a proteome approach indicated that the absence of MapB did not lead to an extensive alteration in OMP abundance, but to a reduction in the relative amounts of a protein subset, including proteins from the Omp25/31 family. Electron microscopy revealed that ∆*mapB* cells exhibit multiple anomalies in cell morphology, indicating that the absence of the TamB homologue in *B*. *suis* severely affects cell division. Finally, ∆*mapB* cells were impaired in macrophage infection and showed an attenuated virulence phenotype in the mouse model. Collectively, our results indicate that the role of *B*. *suis* TamB homologue is not restricted to participating in the translocation of autotransporters across the OM but that it is essential for OM stability and protein composition and that it is involved in cell envelope biogenesis, a process that is inherently coordinated with cell division.

## Introduction

Bacteria of the genus *Brucella* are gram-negative bacteria, responsible for brucellosis, a disease characterized by chronic infections, abortions and infertility in animals, and chronic fatigue in humans^1^. *Brucella* invades and replicates in a variety of host cells such as macrophages, trophoblasts, dendritic and epithelial cells, within a characteristic compartment (“brucellosome”) derived from the endoplasmic reticulum^2^.

The bacterial cell envelope is the major point of interaction between intracellular pathogens and the host and, therefore, the molecules that are part of or are built within it have fundamental roles in the success of infection. The *Brucella* cell envelope, and in particular the outer membrane (OM), exhibits unique characteristics that make these pathogens resistant to most of the host bactericidal agents. Additionally, several evidences indicate that the *Brucella* envelope promotes evasion from innate immunity and is crucial to avoid intracellular destruction^3^. The *Brucella* cell envelope has been subject of numerous studies due to its central role in infection success. *Brucella* OM forms very stable bilayers^4,5^ as several *Brucella* outer membrane proteins (OMPs) maintain hydrophobic interactions with other OM components and/or contain hydrophilic domains that allow their binding to the peptidoglycan^5,6^. In fact, it was proposed that the interaction of the peptidoglycan with the OM results in a higher “OM stiffness” in *Brucella* than in other gram-negative bacteria^4,7^. *Brucella* contains an unconventional non-endotoxic lipopolysaccharide (LPS) that confers resistance to host antimicrobials^5,8^. Both the lipid A and the *core* of *Brucella*’s LPS exhibit a lower number of negative charged groups compared to most LPS from other bacteria^2^. Thus, the physicochemical characteristics of *Brucella*’s OM result in a low permeability (and thus higher resistance) to polycations such as polymyxin B and lysozyme, and a relatively high permeability to hydrophobic compounds^9–11^.

In addition to the OM general features, the cell envelope of bacterial pathogens usually displays adhesive molecules or structures that mediate the adhesion to the extracellular matrix and host cells, including proteins from the autotransporter families which are also in some cases required for full virulence in mice^12–14^. The C-terminal ends of monomeric autotransporters form a 14-stranded antiparallel β-barrel that is inserted into the OM and is essential for the functional (“passenger”) domain to go through the OM^15^. The trimeric autotransporters have a smaller C-terminal translocator domain that shares structural and functional similarities with that of the monomeric ones^16^. Several evidences indicate that the term “auto” actually does not contemplate the translocation mechanism of autotransporters since other proteins are required for their proper secretion. Indeed, the β-barrel assembly machinery (BAM) was found to be required for translocation of several autotransporters^17^. The BAM complex is composed of the essential BamA protein and accessory proteins. BamA belongs to the Omp85 protein superfamily that contains a C-terminal β-barrel domain inserted in the OM, and 5 POTRA (polypeptide-transport-associated) periplasmic domains at the N-terminal region, which were proposed to mediate the initial interaction of the BAM complex with substrate polypeptides^18^. Evidence was presented recently indicating that a new system called Translocation and Assembly Module (TAM) was necessary for the efficient translocation of the Ag43 and p1121 autotransporters in *E*. *coli* and *Citrobacter rodentium*, respectively^19^. The TAM system consists of TamA, another member of the Omp85 superfamily, and TamB, a large protein that is inserted in the inner membrane (IM) by an N-terminal non-cleavable signal peptide; the rest of TamB exhibits an extensive β-helical structure that is immersed in the periplasm^19,20^. The TAM system was not found to be essential in *Gammaproteobacteria*, but its inactivation affected the virulence and the host colonization of *C*. *rodentium* and *Salmonella enterica*^19^. It was recently suggested that a hydrophobic cavity with a β taco fold in TamB may serve as a chaperone to guide the hydrophobic β strands of TAM targets through the periplasm^20^. Proteins from the TamB family were found to be widely distributed in most of the gram-negative bacterial lineages, and surprisingly, even in numerous genera that do not harbor autotransporters^21^. This raises the question of which is the primary role of TamB in the biogenesis of the bacterial cell envelope. Interestingly, other studies suggest that the members of the TamB family from *Aggregatibacter actinomycetemcomitan* (a gammaproteobacterium), *Deinococcus radiodurans* (*Deinococcus*-*Thermus* phylum) and *Borrelia burgdorferi* (a spirochaete) may have a more general role in the OM assembly^22–25^.

While performed an *in silico* analysis to identify *Brucella* autotransporters the BR0049 gene from *B*. *suis* 1330 came out as a possible adhesin with a low similarity to autotransporters. Probably this was due to the abundance of β-helix strands that are predicted along almost the entire protein and also to a β-sheet structure found in the very C-terminus of BR0049 and its orthologues from other *Alphaproteobacteria*^21^. Further *in silico* studies and recent findings on the evolution of the novel TAM machinery^21^ indicated that BR0049 and its orthologues from other *Alphaproteobacteria* encode proteins that are phylogenetically related to members of the TamB family. Evidence presented in this work shows that BR0049 (a TamB homologue) plays roles that go beyond that of participating in autotransporter assembly. We propose that BR0049 is additionally involved in cell envelope biogenesis, in a process that is inherently coordinated with cellular division and that is crucial for cellular integrity.

## Results

### BR0049 is a distant homologue of TamB

The gene annotated as BR0049 in the *B*. *suis* 1330 genome encodes a predicted protein of 1515 amino acids. However, a careful alignment analysis of its upstream DNA region and that of its orthologues in other *Brucella*’s species showed that the start codon of the open reading frame might be located 192 bp upstream the annotated start codon. N-terminal signal sequence prediction (see below) further support this observation. To avoid confusion, we called the corrected ORF as BR0049*. This gene encodes a protein which has 1579 amino acids in length and is highly conserved among the different *Brucella* species (>99% aminoacid sequence identity). Outside *Brucella* spp., its closest homologues with an 80–84% sequence identity are the orthologues of the *Ochrobactrum* genus (another member of the *Brucellaceae* family). In other *Alphaproteobacteria* such as rhizobia, the homologues are similar in size and share 30–50% amino acid sequence identity. Database searches using the DELTA-BLAST program were able to detect phylogenetically related proteins in *Gammaproteobacteria* such as TamB from *Escherichia coli* and *Citrobacter rodentium*^19^, which shared 22% global sequence identity with BR0049. In line with these findings, it was proposed that BR0049* and its orthologues from other *Alphaproteobacteria* would also be part of the TAM system^21^.

**Figure 1:**
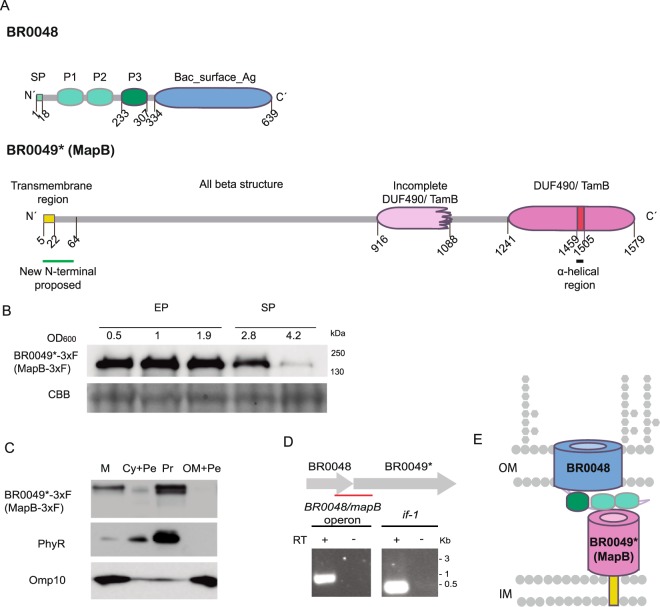
BR0048 and MapB are *B*. *suis* TAM orthologues. (**A**) Scheme of the predicted BR0048 and MapB domain organization. (**B**) MapB-3xFLAG (MapB-3xF) expression along the growth curve. Samples were harvested at the indicated optical densities (at 600 nm) of the exponential (EP) or the stationary (SP) phases. Equivalent total proteins obtained from whole-cell lysates were analyzed by Western blot using a monoclonal anti-FLAG antibody. Cropped blots are shown. (**C**) Determination of MapB-3xF inner membrane localization. Total membranes (M), cytoplasm plus periplasm (Cy + Pe), protoplast (Pr) or outer membrane plus periplasm (OM + Pe) fractions were analyzed by Western blot using antibodies anti-FLAG (upper panel), anti-PhyR as a cytoplasmic control (middle panel) or anti-Omp10 as an OM control (lower panel). MapB-3xF and Omp10 were analyzed on the same blot. The experiment was repeated three times with similar results. Cropped blots are shown. The complete blots from B and C are included in Supplementary Information (Fig. S4). (**D**) The BR0048 and *mapB* genes form an operon. RT-PCR amplified a 700 bp-genomic region (underlined) including coding sequences for both BR0048 and *mapB* or a 162 bp coding region of the housekeeping gene *if-1* as a positive control. Lanes corresponding to reactions performed with (+) or without (−) RT are indicated. (**E**) Scheme of predicted topology of BR0048 and MapB spanning the bacterial cell envelope. Positions of the outer (OM) and inner (IM) membrane are indicated.

Similar to TamB from *Gammaproteobacteria*, protein domain predictions showed that BR0049* contains a DUF490 conserved domain^19^ at the C-terminal region (amino acids 1241–1579) (Fig. 1A). In contrast, the rest of BR0049* diverges from TamB of gammaproteobacteria. BR0049* displays a second and incomplete DUF490 domain (amino acids 916–1088) but with less significance (E-value: 8.57e^−05^) (Fig. 1A), which is not found in *Gammaproteobacteria* TamB. An additional TamB protein feature was found in BR0049* as a β-sheet secondary structure is predicted by the Jpred 4 protein secondary structure server in most of the protein except for an α-helical small region of about 46 amino acids (1459–1505 amino acid region) (Fig. 1A), starting at the same relative position from the C-terminal end of TamB from *E*. *coli*^19^. These observations strongly suggest that the BR0049* protein represents a TamB orthologue although a significant portion of the N-terminal region diverges from the TamB proteins from gammaproteobacteria.

It was previously shown that TamB from *E*. *coli* and *C*. *rodentium* is inserted in the IM through a non-cleavable N-terminal signal sequence but most of the protein is thought to be immersed in the periplasm^19,20,26^. Analysis with the SignalP-4.1 algorithm predicted an N-terminal signal sequence at BR0049* positions 1–21. This sequence overlaps with a probable transmembrane region between positions 5–22 (THTMM 2.0). Therefore, similarly to TamB proteins, the N-terminal end of BR0049* could represent a non-cleavable signal sequence that mediates the insertion of the protein into the IM. Analysis with the Gneg-mPloc program support the hypothesis that BR0049* localizes in both the IM and the periplasm.

To determine the localization of BR0049*, a 3xFLAG tag was fused to the C-terminus of the protein and the presence of the BR0049*-3xFLAG protein in different subcellular fractions was detected with anti-FLAG antibodies. Western blot analysis of total cell extracts showed that BR0049*-3xFLAG is expressed along the exponential and early stationary growth phases, but diminishes at the late stationary phase (Fig. 1B). As an efficient separation of IM and OM has not yet been achieved in *Brucella*, BR0049*-tagged localization was first analyzed in total membranes and in the cytosol fractions. Western blot analysis clearly indicated that BR0049*-3xFLAG localized to the membrane fraction (Fig. 1C). In order to discriminate between the IM and OM, a protoplast fraction (Pr) separated from a OM plus periplasm enriched fraction (OM + Pe) was obtained by treatment with Zwittergent 3–16 followed by centrifugation^27^. Immunodetection with anti-FLAG antibodies showed that the tagged protein localized to the protoplast fraction (Fig. 1C), confirming that BR0049* is associated with the cell IM.

A gene encoding a TamA homologue (BR0048) is found immediately upstream the BR0049* gene (Fig. 1D). TamA represents the OM component of the TAM system^21^ and belongs to the Omp85/TpsB superfamily, whose members were mainly implicated in β-barrel assembly machinery^18^. TamA is inserted in the OM through its C-terminal Bac_surface_Ag domain that exhibits a β-barrel structure, followed in the periplasmic space by a classical POTRA domain and two divergent POTRA domains^26^. A classical POTRA domain (P3) located adjacent to the C-terminal Bac-surface-Ag domain was identified between BR0048 protein amino acids 233–307 (Fig. 1A). In addition, alignment and secondary structure analyses suggest the presence of two other divergent POTRA-like domains (P1 and P2) (Fig. 1A).

It was proposed that *tamA* and *tamB* genes are co-transcribed in an operon^19^. Hence, in order to verify if this organization is conserved in *B*. *suis*, RT-PCR analysis was conducted using total *B*. *suis* RNA and specific primers designed to amplify a genomic region overlapping the 3′end of BR0048 and the 5′end of BR0049. A PCR product of the expected size was obtained, showing that BR0048 and BR0049 genes form an operon (Fig. 1D).

In conclusion, the predicted protein domain organization, subcellular localization and co-transcriptional analyses strongly suggest that BR0048 and BR0049* proteins conform the *Brucella* spp. TAM system (Fig. 1E).

### A BR0049* mutant shows an altered cell surface

**Figure 2:**
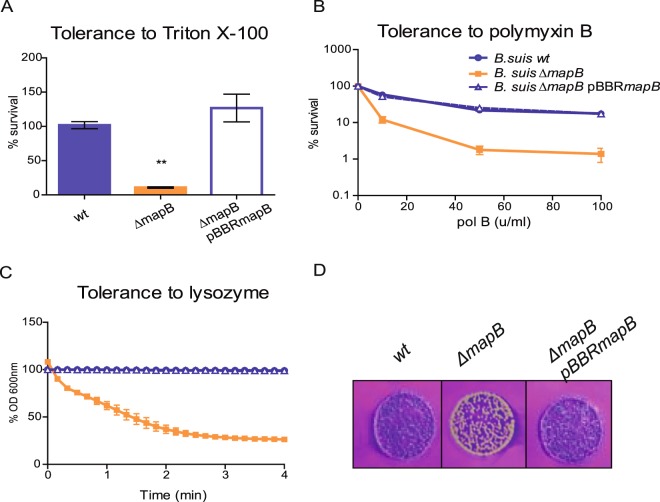
Decreased tolerance to different stresses/compounds demonstrates membrane alteration in the Δ*mapB* mutant. *B. suis* 1330 (wt), Δ*mapB* mutant and the isogenic Δ*mapB* pBBR*mapB* complemented strain were analyzed. (**A**) Bacteria were incubated with or without Triton X-100 and CFU were determined. Tolerance was expressed as the mean percentage of CFU relative to non-treated bacteria. The % survival of each strain relative to the wt is shown. Data were analyzed by one-way ANOVA followed by a Tukey’s *posteriori* test. *Significantly different from the control (wt), (p < 0.05). (**B**) Bacteria were incubated for 60 min at 37 °C with 0 (control), 10, 50 or 100 units/ml of polymyxin B and CFU were determined. Survival was expressed as the mean percentage of CFU relative to non-treated bacteria. (**C**) Bacteria were treated with lysozyme and OD_600_ of the suspension was determined every 10 seconds. Data represent the mean ± standard deviation (SD) of triplicates of one representative experiment out of three independent experiments. (**D**) Crystal violet staining of *B*. *suis* 1330 (wt), Δ*mapB* mutant and the isogenic Δ*mapB* pBBR*mapB* complemented strain.

In order to perform functional studies on the BR0049* protein, we generated a clean deletion mutant of the BR0049* gene by double homologous recombination. As several assays showed that the BR0049* null mutant displayed an altered cell surface (see below) the gene-encoded protein was referred as MapB for membrane altering protein. First, we found that a 10 min preincubation in 0.1% Triton X-100 sharply decreased cell viability of mutant bacteria but not that of wild type cells. Complementation of the mutant with the *mapB* gene cloned into a pBBR1MCS1 plasmid (∆*mapB* pBBR*mapB*) restored resistance to Triton X-100 (Fig. 2A), thus providing phenotypic confirmation of *mapB* deletion. To note, no significant differences in bacterial growth in liquid rich medium were found between the wild type, the ∆*mapB* mutant and the complemented strains (Supplementary Fig. S1). Besides, the ∆*mapB* mutant and the wild type strain did not show statistically significant differences in the sensitivity to different concentrations of H_2_O_2_, 0.67 mM EDTA, 0.01% SDS, 0.1% DOC, porcine serum or acidic pH (4.5) (Supplementary Fig. S1).

*Brucella* is particularly resistant to cationic peptides^5,9^. The ∆*mapB* mutant strain exhibited from 5 to 14-fold higher sensitivity to different concentrations of the cationic lipopeptide polymyxin B than the wild type strain, while the complementing plasmid restored the wild type phenotype (Fig. 2B). *Brucella* species are also relatively resistant to the lytic effect of lysozyme^9^. Remarkably, while the wild-type strain showed a sustained optical density (OD_600_) over time in the presence of lysozyme, the turbidity of the Δ*mapB* culture decreased up to 26% of the initial value after 4 min of incubation due to cell lysis, and the complementing plasmid restored the wild type phenotype (Fig. 2C).

These observations showed that the integrity and typical resistance of the *Brucella* cell envelope to polymyxin B and lysozyme depend to a high extent on MapB, suggesting that the TamB homologue plays in *B*. *suis* a critical role in cell envelope homeostasis.

### *mapB* deletion did not greatly affect the LPS structure and the total fatty acid composition

An increased sensitivity of *Brucella* to polymyxin B has been previously associated to loss of the LPS O-antigen^9,28^. This was not the case for the Δ*mapB* mutants because, firstly, the same as wild type cells, they did not agglutinate in the presence of 0.1% w/v acriflavine. On the contrary, *Brucella ovis*, a rough control that lacks the O-antigen, showed agglutination in the presence of the reagent (data not shown). Secondly, we observed that both the mutant and the wild-type strains exhibited a similar degree of reactivity towards a fluorescein (FITC)-conjugated goat anti-*Brucella* S (smooth) antibody (data not shown). These observations suggest that the O-polysaccharide still remained associated to the cell surface in the Δ*mapB* mutant.

Rough *Brucella* cells devoid of the O-antigen retain more crystal violet compared to their parental strains^29^; *B*. *ovis*, a naturally rough species, retains high amounts of the dye. As expected, we did not observe an increased staining of the Δ*mapB* colonies on Tryptic Soy Agar (TSA) plates in the presence of crystal violet compared to the wild type and complemented strains. On the contrary, while wild type colonies showed an evident violet coloration in the presence of crystal violet, mutant colonies hardly retained the dye. The complementing pBBR*mapB* plasmid restored the phenotype (Fig. 2D). Hence, even though this observation further supports that the Δ*mapB* mutant is not a rough strain, the mutant colony phenotype on crystal violet is consistent with the idea that the Δ*mapB* mutant displays an altered cell envelope.

In order to evaluate whether the Δ*mapB* phenotypes could be attributed to LPS structural differences other than the complete loss of O-antigen, LPSs obtained by SDS-proteinase K treatment were analyzed by silver staining and Western blot. With the exception of some extra bands or bands with stronger signal in the mutant profile, no major differences between the LPS patterns of wild type, mutant and complemented strains were observed by Silver staining (Supplementary Fig. S2). Further analyses by Western blot using anti-O-antigen and anti-R (rough)-LPS monoclonal antibodies, indicated that both the S(smooth)-LPS and R-LPS profiles did not undergo major changes, further confirming that Δ*mapB* is a smooth strain (Supplementary Fig. S2). Again, some extra or stronger bands were observed in the mutant profile (indicated by arrows). To note, we consistently observed by Coomassie Brilliant Blue staining that the LPS from the Δ*mapB* mutant swept along more proteins than that of the wild-type strain (data not shown), suggesting that the extra- or more abundant bands observed in the mutant pattern could be related to this behavior. Association of the LPS from the mutant strain with different proteins could not account for this effect since a similar protein composition was detected by proteomics in both LPS wild type and mutant preparations (data not shown). In fact, 6 members of the Omp25/31 group that were shown to interact with the peptidoglycan^7^, and two other putative OMPs (BR1469 and BRA0921) were associated with the LPS in both the wild type and Δ*mapB* mutant (data not shown). A possible explanation for these observations is that the absence of MapB might result in a weaker interaction of certain OMPs with other envelope structures such as the peptidoglycan or, alternatively, to a stronger OMP interaction with the LPS itself^30^. Interestingly, using LPS preparations obtained by hot-phenol extraction, both the extra bands or bands with stronger signals were not observed in the silver staining Δ*mapB* pattern (Supplementary Fig. S2), suggesting that indeed the subtle differences observed in the profile of crude mutant LPS preparations could be due to a differential interaction of OMPs with envelope structures.

In conclusion, although subtle changes in the LPS structure cannot be ruled out, all our observations indicate that it is unlikely that the cause of the increased sensitivity of the ∆*mapB* mutant to polymyxin B was the lack of the O-antigen or major alterations in the LPS structure.

The cell surface phenotypes of Δ*mapB*, in particular the increased sensitivity to Triton X-100, could be due to changes in the membrane fatty acid composition. These modifications could make the cell more permeable and, therefore, more susceptible to non-ionic detergents. To explore this possibility, membrane fatty acid composition from total lipids was analyzed. Supplementary Table S1 shows no significant differences in the fatty acid amounts and composition of the wild type, mutant and complemented strains. To note, 18:1 and 19:0 cyc should be considered together since, depending on the age of the culture, 18:1 is a direct precursor of 19:0 cyc^31^. This observation shows that the greater sensitivity of Δ*mapB* to Triton X-100, lysozyme and polymyxin B was not due to major changes in membrane fatty acid composition.

### Role of MapB in the insertion of proteins into the OM

**Figure 3:**
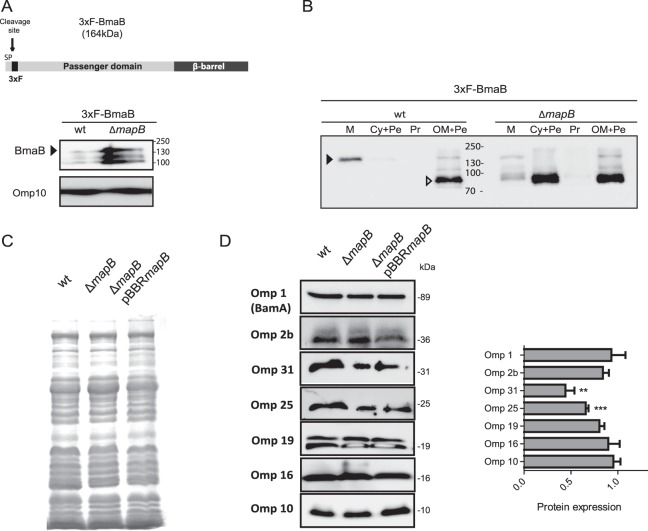
Insertion of a subset of proteins into the OM is altered in the Δ*mapB* mutant. (**A**) Scheme of the predicted 3xF-BmaB domain organization (SP: signal peptide, 3xF: 3xFLAG tag). The arrow indicates the predicted cleavage site. Western blot analysis of *B*. *suis* 1330 strain (wt) whole-cell extracts bearing the pBBR3xFLAG*bmaB* vector (wt-3xF-BmaB), and Δ*mapB* strain with the pBBR3xFLAG*bmaB* vector (Δ*mapB-*3xF-BmaB) performed with anti-FLAG monoclonal antibodies. The arrowhead indicates the predicted molecular weight of the full protein with the FLAG. Anti-Omp10 was used as a loading control. The 3xF-BmaB protein and the loading control (Omp10) were analyzed on the same blot. Cropped blots are shown. The experiment was repeated three times with similar results. (**B**) BmaB subcellular localization. Western blot analysis of 3xFLAG-BmaB was performed with anti-FLAG monoclonal antibodies on total membranes (M), cytoplasm plus periplasm (Cy + Pe), protoplast (Pr) and outer membrane plus periplasm (OM + Pe) fractions in the wt-3xF-BmaB and Δ*mapB-*3xF-BmaB strains. The black arrowhead indicates the predicted molecular weight of the full protein with the FLAG (164 kDa); the white arrowhead indicates the 85 kDa major protein fragment. (**C**) SDS-PAGE and Coomassie Brilliant Blue staining of whole-membrane fractions. (**D**) Western blot of whole-membrane fractions with anti-Omp1, Omp2b, Omp31, Omp25, Omp19, Omp16, or Omp10 antibodies. Quantification of OMPs abundance from three independent experiments was determined relative to Omp10 as loading control, and the relative OMP abundance in the Δ*mapB* mutant was represented as protein expression relative to the wt strain. According with the target molecular weight the membranes were eventually cut horizontally before blotting. In C and D, the samples are derived from the same experiment and processed in parallel. In A and D, the images were cropped horizontally for presentation. Representative original blots are included in Supplementary Information (Fig. S5). Data were analyzed by one-way ANOVA followed by a Tukey’s *posteriori* test. *Significantly different from the control (wt), (p < 0.05).

As mentioned above, TamB proteins from *E*. *coli* and *C*. *rodentium* were shown to be required for efficient translocation of autotransporters into the OM^19^. To assess whether a similar function could also be ascribed to MapB, we analyzed the presence of the *B*. *suis* monomeric autotransporter adhesin BR2013 (BmaB) (Bialer *et al*., unpublished data) in whole cell extracts and in different subcellular fractions of wild-type and Δ*mapB* strains. A plasmid construct harboring an N-terminal 3xFLAG translation fusion of BmaB at the signal peptide cleavage site (Fig. 3A) was generated and the presence of the tagged protein was analyzed by Western blot using anti-FLAG antibodies. As expected, the presence of a protein band with the predicted molecular weight of 164 kDa as well as two other associated protein bands were observed in wild-type whole cell extracts (Fig. 3A). Intriguingly, increased levels of 3xFLAG-BmaB were consistently observed in whole cell extracts of the Δ*mapB* mutant (Fig. 3A). We then determined the subcellular distribution of the tagged autotransporter in both cellular backgrounds. Given the difficulty in separating the OM from the IM in *Brucella* cells, total membranes (M) and soluble (Cy + Pe, cytoplasm plus periplasm) fractions were analyzed by Western blot using the anti-FLAG antibodies (Fig. 3B). As expected, a major 164 kDa band was mainly observed in total membranes in the wild type background. In contrast, although a low amount of the entire protein was recovered in the Δ*mapB* total membrane fraction, high levels of it were associated with the soluble fraction (Cy + Pe) as an 85 kDa fragment. Proteolytic breakdown was not prevented by the addition of a protease inhibitor cocktail (Fig. 3B).

To further study the autotransporter localization in both genetic contexts, the protoplasts (Pr) and the OM-periplasm (OM + Pe) enriched fractions obtained by Zwittergent 3–16 treatment (see Materials and Methods) were also analyzed by Western blot. As expected, in the wild-type strain the 3xFLAG-BmaB protein localized to the OM + Pe fraction, though in this case mainly as a protein fragment of approximately 85 kDa, strongly suggesting that the autotransporter is particularly sensitive to the fractioning procedure. Similarly, in the Δ*mapB* mutant a protein fragment of the same molecular weight (85 kDa) was also detected in the OM + Pe fraction, though in higher amounts than in the wild-type strain (Fig. 3B). Therefore, taking together the protein patterns of all subcellular fractions obtained by both fractioning methods, it may be concluded that in the wild type background BmaB is located in the OM. On the other hand, although some autotransporter molecules seemed to reach the OM in the ∆*mapB* mutant, comparison of protein amounts in the different protein fractions indicated that most of BmaB was recovered in the periplasm.

The altered cell envelope homeostasis of Δ*mapB* could hardly be explained by a defect in the efficiency of OM insertion of proteins with adhesin activity. Thus, a plausible hypothesis was that MapB is involved in the OM translocation of either a broad range of substrates or, alternatively, of a specific subset of proteins, some of which would be important for OM assembly. To evaluate these alternatives, the presence of a variety of different OMPs in total membranes of the wild type, Δ*mapB* and the complemented strains was analysed by Western blot using specific antibodies. We assumed that OMPs not efficiently translocated into the mutant OM would be underrepresented in the samples. The following OMPs were chosen for this analysis: Omp1 (BamA)^32^, Omp2b (an oligomeric porin with β-barrel structure)^6,33^, Omp31 and Omp25 (group 3 of *Brucella* OMPs, with β-structure, associated with the peptidoglycan and exposed on the surface)^5,6,34^ and Omp19, Omp16 and Omp10 (all lipoproteins with a hydrophilic domain and possible binding to the peptidoglycan)^5,35^. Coomassie Brilliant Blue or silver staining profiles of total membranes from the wild type, the ∆*mapB* mutant and complemented strains showed no major differences (Fig. 3C), indicating that the mutant did not undergo extensive changes in the OMP composition. As to the Western blot, no significant differences were found between the wild type and the mutant in the protein levels of Omp1 (BamA), the Omp2b porin or the Omp19, Omp16 and Omp10 lipoproteins (Fig. 3D).

Analysis of group 3 of OMPs using anti-Omp25 or anti-Omp31 antibodies did not show discrete bands but a smearing across the lanes in both the wild type and the mutant membrane preparations (Supplementary Fig. S3). Interestingly, membrane treatment with 5 mg/ml lysozyme, followed by Western blot analysis resulted in the observation of discrete bands in both genetic backgrounds (Fig. 3D). This is in agreement with the strong peptidoglycan association that has been previously proposed for this group of OMPs^7^. It is noteworthy that antibodies used probably recognize more than one member of the Omp25/31 protein family^34,36,37^. A 50% decrease in levels of membrane-associated Omp25 and Omp31 proteins was observed in the ∆*mapB* mutant in comparison with wild type cells. The complemented strain showed restored protein levels (Fig. 3D). In line with this observation, it was previously proposed that a tight balance of the OMPs from group 3 is essential for *Brucella* OM integrity^5,38^. Reduction in Omp25 and Omp31 amounts was not due to a decrease in protein expression since Western blot analysis of whole cell extracts did not show differences in those OMP amounts between the wild type and mutant backgrounds (Supplementary Fig. S3).

For a more global analysis, total membrane proteins from the wild type, Δ*mapB*, and complemented strains were analyzed in triplicate by LC-MS/MS using the Orbitrap technology. Label-free quantification analysis showed a similar abundance of most of detected proteins in the three strains, further indicating that the absence of MapB did not induce a general decrease (or change) in the relative amounts of membrane proteins. It should be stressed that our analysis was restricted to proteins detected in all three replicates of both strains. Furthermore, in no case proteins detected in the three wild type cell replicas appeared to be absent from one or more of the three mutant ones. In line with Western blot studies, similar abundance of Omp1 (BamA), of the Omp2b porin and of the Omp16 lipoprotein was observed in the wild type and the mutant cells (Table 1). Furthermore, several other OMPs, including proteins with β-barrel structure such as BepC^39^ and proteins from group 3 of OMPs (Omp25 and Omp25b) were detected and showed similar abundances in both strains (Table 1).

**Table 1:** OMPs detected by nano LC-MS/MS.

ID	Gene code	OMP/Description	Structural features	*p*-value	Fold change
Q45689	BR0701	Omp25	β-barrel	0.57	—
P0DI95	BR0639	Omp2b	β-barrel, trimer, porin	0.15	—
P0A3U5	BRA0423	Omp31	β-barrel, trimer^a^	0.033	-6.4
A0A0H3G433	BR1622	Omp31b	β-barrel	0.014	-7.4
A0A0H3G4S3	BR0119	Omp25c	β-barrel	0.0034	-5.2
A0A0H3G234	BR0440	Uncharacterized OMP (MliC^b^)		0.7	—
A0A0H3G415	BR1154	BamA	β-barrel and periplasmic domain	0.046^c^	—
P0A3S8	BR1695	Omp16	Lipoprotein, monomer	0.63	—
A0A0H3G313	BR0971	Omp25b	β-barrel	0.78	—
A0A0H3G2L4	BR0708	Uncharacterized OMP (DUF992^d^)	0.047	—	

^a^Trimeric structure was suggested by the ability to form SDS-resistant oligomers^4^.

^b^MliC, Membrane-bound lysozyme inhibitor of C-type lysozyme.

^c^The *p*-value is in the limit of significance. However, Western blot analysis showed similar BamA abundances in the wild type and mutant backgrounds.

^d^DUF992, conserved domain of unknown function.

In agreement with Western blot results shown in Fig. 3D, abundance of both Omp31 members (BRA0423 and BR1622)^34,37^ were diminished in the mutant. In addition, the levels of Omp25c (BR0119)^37^ were also reduced compared to the wild type cells. This may account for the decrease in the signal observed by Western blot using anti-Omp25 antibodies (Fig. 3D); however, we cannot rule out the contribution of Omp25d to this result since the abundance of this protein was under the good confidence detection limit.

The abundance of four proteins with a predicted subcellular localization other than the OM was also decreased in Δ*mapB* in comparison with the wild-type and complemented strains (Supplementary Table S2). The levels of two IM proteins were reduced in the mutant: an oligopeptide ABC transporter related to β-lactam resistance (BRA0535) and a putative lipoprotein anchoring transpeptidase (BR0564) from the ErfK/SrfK family^40^. The homologue of the latter in *Agrobacterium tumefaciens* (Atu0845) was proposed to play a major role in peptidoglycan crosslinking in the growing pole during the cell division process^41^. In addition, the levels of BR2114 a protein that according to the Gneg-mPLoc server has a predicted periplasmic localization and that harbors a DUF2852 domain of unknown function, were reduced in the mutant. Finally, the abundance of a cytoplasmic protein from the DegT/DnrJ/EryC1/StrS family (BR1601), whose members were involved in 4-amino-6-deoxy-monosaccharide D-perosamine synthesis, was decreased in Δ*mapB*^42^ (Supplementary Table S2).

Taken together, these results demonstrate that the mutant phenotypes are not due to a generalized change in OMP abundance. However, our observations strongly suggest that MapB is required for the correct localization or stability in the OM of a subset of OMPs, including the BmaB autotransporter and members of group 3 of OMPs (Omp25/31 family). The role of MapB in OM insertion of additional OMPs cannot be completely ruled out since the comparative analysis between the wild type and the mutant membrane proteomes was carried out under very stringent conditions. The relative reduction of other proteins that, according to their amino acid sequences cannot be predicted to localize to the OM, might be related to indirect effects. For instance, the presence of some of those proteins in their final destinations could be dependent on their interaction with OMPs whose stability in the OM is MapB dependent.

### The absence of MapB leads to aberrant cellular division

**Figure 4:**
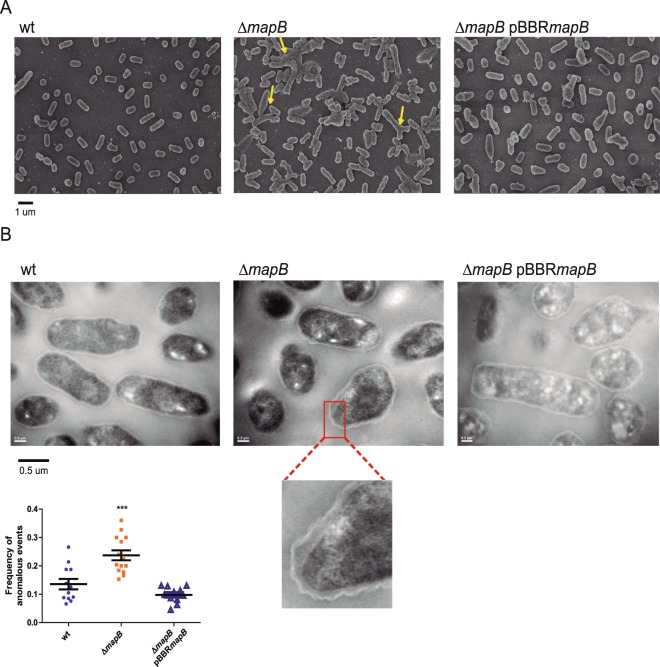
Δma*pB* cells show aberrant cell division. (**A**) *B*. *suis* 1330 (wt), Δ*mapB* mutant and the isogenic complemented Δ*mapB* pBBR*mapB* bacteria were grown in rich medium up to early stationary phase, processed for SEM analysis and observed under Carl Zeiss NTS SUPRA 40. (**B**) TEM analysis of bacterial ultrathin sections of strains shown in (**A**). The inset shows a representative section of the OM detachment observed in the Δ*mapB* mutant. Quantification of cells with detached OM. The number of events were registered and plotted as a frequency of occurrence. Data were analyzed by one-way ANOVA. ***Significantly different from the wt (p < 0.0001).

It has been proposed that the TAM system works through the interaction between the periplasmic domains of TamB IM and TamA OM proteins. This topology is itself suggestive of a function for the TAM complex related with cell envelope biogenesis. In addition, the low stability of the ∆*mapB* cell envelope under different stress conditions suggests that MapB is crucial for cell envelope homeostasis. Therefore, to evaluate changes in the Δ*mapB* surface and cell morphology we performed Scanning Electron Microscopy (SEM) and Transmission Electron Microscopy (TEM) analyses of stationary-phase bacteria. Surprisingly, SEM analysis showed that, unlike the typical small coccobacillus morphology of *Brucella*, *mapB* deletion led to an increase in cell size, the formation of ectopic septa, several “Y shaped” bacteria, and other aberrant cell morphologies (Fig. 4A). These observations strongly suggest that MapB absence causes defects in cell division. The complemented strain showed a high proportion of cells looking like the wild-type strain although some bacteria showed atypical shapes, thus indicating that this last phenotype was not completely restored. TEM analysis showed that in a low but significant number of cells the Δ*mapB* OM was slightly more separated from the IM with focal looser appearance than in the wild-type strain. The complementing plasmid restored this last phenotype (Fig. 4B).

*B*. *abortus* and *B*. *melitensis* like other bacteria release blebs or outer membrane vesicles (OMVs) to the extracellular medium^43,44^. OMVs are produced in Gram-negative bacteria by OM bulging carrying periplasmic soluble content. It was proposed that while bacteria grow and recycles their OMs, cell walls tends to release more OMVs mostly at the division septa. Besides, it is known that mutants in OMPs linked to the peptidoglycan layer, such as OmpA, Lpp, TolB and Pal in other bacterial genera increase the blebbing process. Thus, we wondered whether the multiple ectopic septa and the decrease in the abundance of OMPs that interact with the peptidoglycan observed in Δ*mapB* might lead to OMV overproduction and/or augmented protein content associated with these vesicles. To test this suggestion, wild type and mutant cells were grown to the exponential phase and OMV fractions that were obtained by ultracentrifugation of cell-free supernatants were analyzed by LC-MS/MS. Very few proteins were detected in the wild type OMV fraction, suggesting a low production of OMVs by *B*. *suis* 1330 under the culture conditions employed. On the other hand, the OMV fraction recovered from the Δ*mapB* strain revealed a high number of proteins (around 40). Almost 60% of detected proteins corresponded to OMPs and periplasmic proteins, including multiple members of the Omp25/Omp31 protein family, Omp2b, BamA, TolB and periplasmic components of ABC transporters (Supplementary Table S3). Thus, the *mapB* mutant tends to release higher amounts of OMV-associated proteins than the wild type cell. Although it cannot be discarded that part of the material recovered comes from detachment of the OM and leakage of periplasmic content, the phenotype observed further confirmed the fragility of Δ*mapB* cell envelope and suggests that the blebbing process is somehow altered or deregulated in the mutant.

It may be concluded that lack of MapB leads to severe defects in the cell division process. In addition, it seems that the absence of MapB affects the OM association with the cell wall, thus leading to higher levels of OMV-associated proteins.

### MapB is required for initial survival in macrophages and virulence in the mouse model

**Figure 5:**
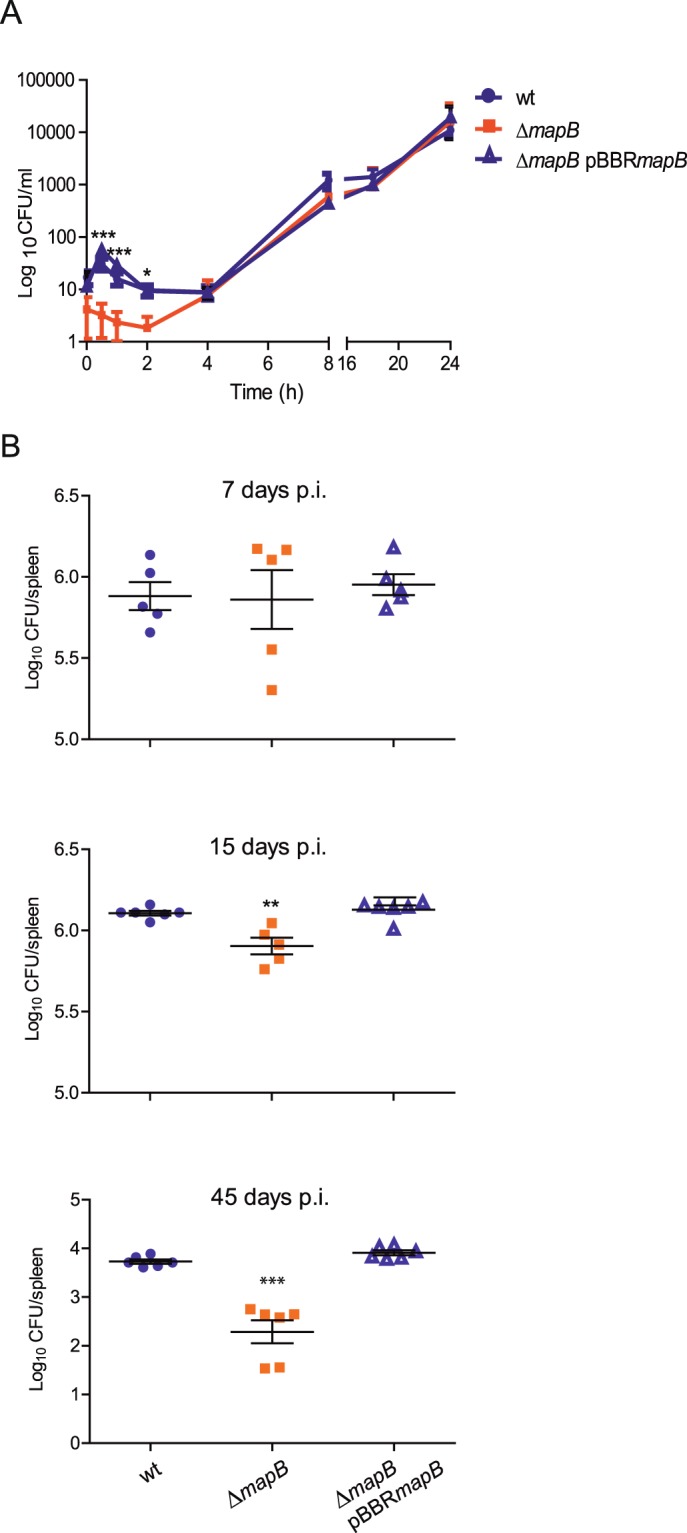
MapB contributes to intracellular survival in macrophages and full virulence in mice. (**A**) Bacterial intracellular replication in J774A.1 murine macrophages. At 0 h (control), 0.5 h, 1 h, 4 h, 8 h, 18 h and 24 h bacteria were recovered from macrophages and plated for CFU count. Results are expressed as Log_10_ of CFU/ml. Data represents the mean ± standard deviation (SD) of triplicates. A representative experiment out of three independent experiments is shown. (**B**) Role of MapB in *B*. *suis* virulence in mice. BALB/c mice were inoculated by intragastric delivery with *B*. *suis* 1330 (wt), Δ*mapB* or the complemented Δ*mapB* pBBR*mapB* strains. 5 mice per group were sacrificed at 7, 15 and 45 days post infection (p.i.). CFU recovered from spleens homogenates were counted and expressed as the Log_10_ CFU per spleen. Data were normalized to t = 0 h and significance was analyzed by one-way ANOVA followed by Tukey’s *posteriori* test. The experiment was performed twice with similar results. *Significantly different from wt (p < 0.05); ***Significantly different from wt (p < 0.0001).

To evaluate if MapB plays a role in the virulence process, we studied the internalization and intracellular replication of the wild type, the complemented and the Δ*mapB* mutant strains in J774.1 murine macrophages. As shown in Fig. 5A, deletion of *mapB* significantly affected the intracellular CFUs, particularly at early stages (30 min to 8 h post infection; p.i.). However, at later stages, bacteria that managed to survive replicated at rates similar to those of wild type cells. These results suggest that Δ*mapB* exhibits a defect in internalization and/or survival during the first stages of macrophage infection but not at later stages.

To further examine the role of MapB in the infection process, we performed *in vivo* studies using the mouse model. To this end, groups of five mice per strain were infected by intragastric inoculation^45^ and splenic infection was evaluated at 7, 15 and 45 days p.i. At 7 days p.i. there were no significant differences between the wild type, the mutant and complemented strains. However, at 15 and 45 days p.i. the splenic infection by ∆*mapB* cells was significantly reduced, by 0.4 log and 1.45 log respectively, compared to the wild-type strain (Fig. 5B). In all cases, the complemented strain harboring the pBBR1*mapB* plasmid showed a splenic CFU count similar to those of wild type cells. These results indicate, therefore, that MapB is required for full virulence of *B*. *suis* in mice infected through the oral route.

## Discussion

**Figure 6:**
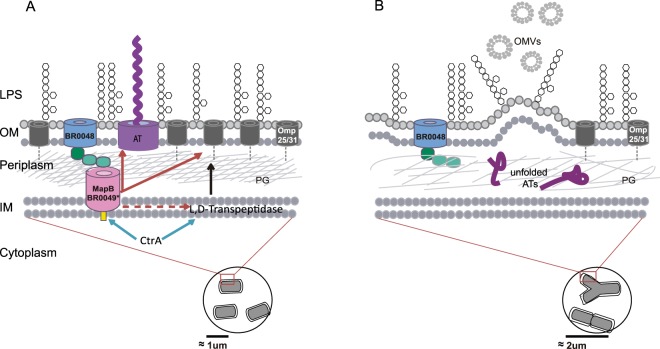
Model for the role of TAM in *B*. *suis*. Bottom circles contain either the wild type (**A**) or the Δ*mapB* mutant (**B**) bacteria, highlighting the altered cell division occurring in Δ*mapB* cells. Only the principal cell envelope components are shown. (**A**) Omp25/31 proteins are represented in black and the BmaB autotransporter in purple. Red solid arrows indicate that the proper translocation of both BmaB and Omp25/31 proteins depends on MapB. The red broken arrow represents an indirect effect on t L,D-transpeptidase abundance. Blue arrows indicate a direct regulation of BR0048/*mapB* operon and the L,D-transpeptidase gene by CtrA, the master regulator of cell division^48^. The black arrow symbolizes the role proposed for L,D-transpeptidase on peptidoglycan crosslinking. (**B**) Δ*mapB mutant* model representing the cell envelope alterations in the absence of MapB: accumulation of unfolded BmaB in the periplasm, decrease in Omp25/31 protein abundance in the OM and an indirect negative effect on L,D-transpeptidase abundance that may contribute to the OM looser appearance, a relaxed peptidoglycan and an overproduction of OMVs containing high levels of protein cargo. OM: outer membrane; IM: inner membrane; PG: peptidoglycan; ATs: autotransporters; OMVs: outer membrane vesicles.

The role(s) of TamB family proteins in the bacterial physiology is poorly understood. In this work we provide information on the role MapB, the *B*. *suis* TamB homologue, in cell envelope biogenesis. The model for this role arising from our observations is depicted in Fig. 6 and will be further discussed below. Our initial observation on the Δ*mapB* mutant marked sensitivity to Triton X-100 suggested that MapB could have a fundamental role in envelope stability. To better understand the impact of MapB absence on cell surface features, the Δ*mapB* mutant was exposed to different stress conditions. We found that bacteria lacking MapB lost the typical brucellae resistance of to both polymyxin B and lysozyme^5,9^, thus indicating that the cell surface characteristics that allow *Brucella* to overcome the deleterious effects of those agents were MapB dependent. Further evidence showed that Δ*mapB* phenotypes were not attributable to evident changes in LPS structure, which is considered a key surface structure that makes *Brucella* resistant to several host antimicrobials, including polymyxin B^9,46^.

Hence, the question was which is the process mediated by MapB (and its putative partner BR0048), whose deficiency produces profound alterations in cell envelope stability. Selkrig and co-workers proposed that TAM is involved in the translocation and assembly of certain proteins, specifically proteins from the autotransporter family^19^. We indeed found that Δ*mapB* cells, instead of targeting the BmaB autotransporter to the OM tend to accumulate BmaB in the periplasm (Fig. 6B). However, the Δ*mapB* phenotypes could not be simply explained by a defect in autotransporter translocation since the functions of this protein family in *Brucella* are restricted to bacterial adhesion to the extracellular matrix (ECM) and host cells^12–14,47^ (Bialer *et al*., unpublished). Besides, neither single mutants nor some double mutants of *B*. *suis* autotransporter genes showed altered Triton X-100 or lysozyme tolerance (data not shown), strongly suggesting that MapB could not be involved solely in autotransporter translocation efficiency. Therefore, a reasonable hypothesis was that the *Brucella* TamB homologue could be involved in the translocation of a larger subset of OMPs, some of which would be important for OM homeostasis. Western blot analysis using specific antibodies against several OMPs and a membrane proteome approach showed that the relative abundance of OMPs from the Omp25/31 family was impaired in the MapB-deficient mutant. Interestingly, it has been previously proposed that a tight balance of proteins from this family is necessary for OM stability, and evidence was reported showing that Omp25/31 proteins strongly interact with both the LPS and the peptidoglycan, thus allowing a higher OM and envelope stiffness^5,38^. Therefore, changes observed in the relative abundance of proteins of the Omp25/31 family could account, at least in part, for the OM instability of MapB-deficient cells (Fig. 6B).

The Δ*mapB* mutant is viable under normal culture conditions but the observation of aberrant cell morphologies indicate that cell division, cell elongation or both are severely altered in the mutant. This implies that the cell envelope biogenesis process mediated by TAM in *Brucella* must be coordinated with cell division. Supporting this hypothesis, a recent analysis by chromatin immunoprecipitation followed by deep sequencing (ChIP-seq) showed that the *B*. *abortus* promoter of the BR0048 and *mapB* orthologues is one of the multiple targets of CtrA, a global regulator of cell division^48^ (Fig. 6A). Interestingly, it was shown that, in addition to recognizing promoters of genes involved in cell cycle progression, CtrA binds promoters of genes encoding proteins that participate in OM biogenesis. A conditional *ctrA* mutant displayed elongated and branched morphologies. These same morphologies were also detectable inside HeLa cells and bacteria with abnormal shapes were able to traffic to the endoplasmic reticulum niche in host cells. Of note, CtrA depletion altered OM composition, in particular Omp25. Thus, some of the phenotypes of the CtrA-depleted cells are reminiscent to those observed in the ∆*mapB* mutant, including the ability to reach the replication niche since, although the *ΔmapB* mutant presented cell division defects, it was able to replicate at later infection stages within host cells (see below). An attractive hypothesis is that MapB somehow temporarily assists CtrA in cell cycle regulation. Further studies will be necessary to confirm the regulation of the BR0048-*mapB* operon by CtrA and the interplay between this last protein and MapB during the cell cycle.

Other few proteins with a predicted subcellular localization other than OM were reduced in the Δ*mapB* strain. The decrease in the abundance of those proteins might be due to indirect effects of the *mapB* mutation on gene expression or on protein stability. The observation that a putative L,D-transpeptidase of *B*. *suis* (BR0564) was diminished in the mutant is interesting since this protein is related to peptidoglycan crosslinking. The L,D-transpeptidases catalyze the 3,3-cross-links between two m-Dap residues thus allowing the linkage of m-Dap residues with the peptidoglycan mesh (Fig. 6A). It has been reported that the *A*. *tumefaciens* orthologue (Atu0845) was crucial for polar growth during cell division^41^. Besides, the *B*. *abortus* homologue (BAB1_0589), and other five genes coding for L, D transpeptidases, are targets of the cell cycle regulator CtrA (see above)^48^. Therefore, decrease in the BR0564 abundance could contribute to the looser appearance of the OM (Fig. 4). It will be interesting to analyze the crosslinking grade of muropeptides in the Δ*mapB* cell wall.

Numerous reports have shown that Gram-negative bacteria, including *Brucella* spp., spontaneously release OMVs during normal growth and different biological roles for those vesicles have been proposed^43,44,49^. Very few proteins were detected in the OMV fraction recovered from the extracellular medium of *B*. *suis* wild-type cells grown up to the exponential phase under normal conditions. In contrast, a considerable number of proteins were detected in the OMV fraction of Δ*mapB* cells, and about 60% of them corresponded to OMPs and periplasmic proteins. This observation suggests that defects in cell envelope homeostasis in Δ*mapB* cells favors OM vesiculation or, alternatively, leads to leakage of periplasmic proteins and release of OM fragments. It has been proposed that OMVs are generated by outward bulging of the OM in areas where proteins linking the OM to the peptidoglycan layer are absent. Furthermore, a role for OM-peptidoglycan linkages in vesicle production has been suggested^50^. It has been shown that cells in the growth exponential phase, which is under a high cell division rate, mainly produce OMVs at the division septa^51,52^. Furthermore, mutants displaying a compromised OM showed a hypervesiculating phenotype^51,53^. Based on these findings, a plausible hypothesis is that a weakening of the OM interaction with the peptidoglycan (like those mediated by proteins from the Omp25/31 family) in the ∆*mapB* mutant might result in the production of a higher number of vesicles (Fig. 6B). However, it should be noted that TEM analysis did not allow us to confirm this hypothesis due to the difficulty in quantifying OMVs. In any case, our observations support the idea that the OM homeostasis is impaired in the ∆*mapB* mutant. Another interesting (although indirect) observation is that the mutant LPS tends to drag more OMPs than that from wild type and complemented cells. Again, an impaired interaction of some OMPs with other components of the cell envelope, such as the peptidoglycan, might explain this observation.

It is possible that in *Brucella*, and other closely related bacteria, TamB proteins could play a role different from that previously described for *E*. *coli*, *C*. *rodentium* and *S*. *typhimurium* (all *Gammaproteobacteria*) TamB proteins^19^. Hence, the question arises as whether TamB proteins from different classes within the Proteobacteria phyla have evolved according to their life style to fulfill different functions related to the cell envelope biogenesis. In this regard, it is worth mentioning that in addition to the characteristic C-terminal DUF490 domain that is present in all TamB proteins, MapB harbors an extra (although incomplete) DUF490 domain (Fig. 1). This fact supports the idea of functional differences between TamB proteins of the Proteobacteria phylum. Interestingly, many phyla harboring a TamB homologue do not encode for autotransporters and in some phyla TamB is genetically associated with a BamA homologue, strongly suggesting a functional divergence in the evolution of TamB and its partners^21^. Moreover, mutants defective in *A*. *actinomycetemcomitans* (a gammaproteobacteria) and *D*. *radiodurans* TamB homologues (MorC for the former) showed that their cell envelopes were compromised^22,25,54^. These evidences support the hypothesis of a functional divergence between TamB homologues from different phyla.

*Brucella* cell envelope is more resistant to non-ionic detergents than that from *E. coli*^55^. The increased sensitivity to Triton X-100 (a non-ionic detergent) of the ∆*mapB* mutant might be explained by direct or indirect effects on fatty acid composition. However, we did not observe major differences in the amounts of membrane fatty acids. Nevertheless, modifications of less abundant fatty acids such as the very long chain fatty acids (VLCFAs) that substitute the LPS lipid A in *Brucella* cannot be ruled out^46,56^. Further structural studies on lipid A using more sensitive methods will be necessary to evaluate if the absence of MapB affects (directly or indirectly) lipid A VLCFA acylation. In addition, a low proportion in *Brucella* spp. OM phosphatidylethanolamine (PE) content has been related to polymyxin B resistance, and increased amounts of PE led to a sensitive phenotype to this reagent^57^. Therefore, it would also be interesting to analyze whether *mapB* mutation affects the phospholipid content.

The Δ*mapB* colony phenotype in the presence of the crystal violet dye was puzzling. It seems that the mutant is almost unable to retain the dye. It is known that the crystal violet dissociates into ions in solution and that the cation is responsible for bacterial staining upon its interaction with negatively charged components of the cell wall, including the LPS and the peptidoglycan. The reason for the mutant phenotype is unknown but it clearly implies critical alterations in cell envelope properties. The inability of the ∆*mapB* mutant cells to retain the dye could be explained either by a reduction in the cell wall negative charges or, based on evidence discussed so far, a weakening of interactions between different components of the cell envelope.

What has been discussed so far suggests that in *Brucella*, TAM might be necessary for a stable interaction of the OM with other components of the cell envelope, such as the peptidoglycan. This function might be fulfilled by aiding the anchoring of OMPs to the peptidoglycan and/or via other unknown mechanism. In addition of OMPs and lipoproteins, OM anchorage is performed by the Tol–Pal complex. The OM invagination occurring upon formation of the septum during cell division is believed to be aided by the Tol-Pal complex, which anchors the OM to the IM and contributes to the OM interaction with the peptidoglycan^58^. Bacteria defective in any Tol-Pal system component become susceptible to several toxic compounds (including detergents), exhibit delayed OM invagination and form high amounts of OMVs at constriction sites and cell poles^58–60^. In addition, the TAM system connects the IM to the OM through TamA-TamB interactions^19,26^, and apparently in *B*. *suis* it is necessary for cell envelope integrity and normal cell division. Therefore, the *Brucella* TAM might behave as another subcomplex that ensures cell envelope homeostasis during cell division.

We showed that MapB is required for efficient invasion and/or survival at initial stages of macrophage infection and also for bacterial persistence in mice. This is not surprising judging for the pleiotropic phenotype of Δ*mapB*. Furthermore, it has been proposed that the cell cycle and virulence of *B*. *abortus* are coordinated^61,62^. Thus, the linkage between MapB and cell division implies that bacteria lacking *mapB* will necessarily be affected in the efficiency of some virulence mechanisms. Furthermore, bacteria in the G1 cell cycle stage (newborn) are more infectious than their counterparts in S or G2 phases^62^. Hence, reduction in CFU recovered from macrophages infected with the ∆*mapB* mutant during the first hours after infection might indicate a lower proportion of infective newborn cell type at the onset of macrophage infection. Defects in cell division (and differentiation) of bacterial cells lacking MapB could be responsible for a negative effect in the generation of the infective cell type. For the same reason, reduction of infective cell species of Δ*mapB* might lead to a decrease in the infection efficiency of neighboring cells and finally in the spread of the infection. This hypothesis could explain the attenuated phenotype of the Δ*mapB* mutant in the mouse model. On the other hand, the instability of certain OMPs in the OM that represent in themselves virulence factors could also lead to impaired adhesion and survival in cell cultures and to an attenuated phenotype in mice. The absence of MapB might also affect bacterial survival during the first hours of mammalian cell infection. Reduced bacterial viability might be explained by defects in bacterial cell envelope homeostasis of Δ*mapB* mutant cells leading to loss of the cell envelope determinants that protect *Brucella* cells against host antimicrobials.

To conclude, in this work we provide compelling evidence that the *B*. *suis* TamB homologue (MapB) is highly relevant for outer membrane composition and stability. We propose that the TAM system in *Brucella*, and possibly in its close relatives, plays a determinant role in cell envelope assembly and in its coordination with cell division.

## Methods

### Bacterial strains, cell culture and media

*Escherichia coli* strains used in this study (DH5α and S17-1) were grown at 37 °C in Luria-Bertani (LB) medium. Antibiotics were added when needed: ampicillin 200 µg/ml, chloramphenicol 50 µg/ml, and tetracycline 5 µg/ml. *Brucella suis* 1330 wild-type (ATCC 23444) and the derivative strains were grown at 37 °C in rich medium Tryptic Soy Broth (TSB, Bacto^TM^) or in Tryptic Soy Agar (TSA, Bacto^TM^). Antibiotics were added when needed: chloramphenicol 6 µg/ml, kanamycin 50 µg/ml and nalidixic acid 10 µg/ml. All *Brucella* strains used in this study were manipulated in a biosafety level 3 laboratory. All *E*. *coli* and *B*. *suis* strains used in this study are listed in Supplementary Table S4. J774A.1 murine macrophages cell line (ATCC® TIB-67™) were cultured in RPMI media (GIBCO), supplemented with 5% fetal calf serum (PAA) and grown at 37 °C in a 5% CO_2_ atmosphere.

### Molecular techniques

All DNA manipulations were carried out using standard procedures. All plasmids and primers used in this study are listed in Supplementary Tables S4 and S5, respectively.

#### ∆mapB strain construction

PCR primers containing restriction enzymes recognition sites were designed to amplify the flanking regions of *mapB* (BR0049*). Primers ∆mapB_F1 and ∆mapB_R1 amplify a region of 306 bp upstream *mapB*, and primers ∆mapB_F2 and ∆mapB_R2 a region of 391 bp downstream *mapB*. The generated PCR fragments were purified, digested with the corresponding restriction enzymes, ligated together and cloned into the pK18mobsacB mobilizable suicide vector (Km^R^)^63^ obtaining the pKmobsacBΔ*mapB* plasmid. This plasmid was transformed into *E*. *coli* S17-1 and then conjugated with the *B*. *suis* wild type (wt) strain (Nal^R^). Double recombinant clones were selected (Nal^R^, Km^S^, sucrose^R^). The clean deletion of the entire open reading frame (ORF) was confirmed by PCR, and the absence of *mapB* gene was confirmed by RT-PCR.

#### Complementation of ∆mapB strain

The *mapB* gene was cloned into a broad-host-range vector, pBBR1MCS-1. A region of 5,284 bp containing 706 bp upstream *mapB* start codon, the *mapB* ORF and 30 bp downstream the stop codon was amplified using primers FComp and RComp. The amplified product was cloned into the pBBR1MCS-1 mid-number-copy plasmid^64^ obtaining the pBBR*mapB* plasmid, which was conjugated into *B*. *suis* Δ*mapB* strain. Complemented clones (*∆mapB* pBBR*mapB*) were selected (Cm^R^) and confirmed by PCR.

#### Confirmation of gene expression and operon organization

Total RNA was isolated from *B*. *suis* wt using the Illustra RNAspin Mini Isolation Kit (GE). Subsequently, reverse transcription was performed with SuperScript III First Strand Synthesis System (Invitrogen) following the manufacturer^'^s instructions. In order to analyze gene expression by RT-PCR, different primers were used: FRTmapB and RRTmapB for *mapB* (BR0049); FRT48 and RRT48 for BR0048; and FRT50 and RRT50 for BR0050. To analyze if BR0048 and BR0049* form an operon, we conducted RT-PCR using the following primers: RtOpF and RtOpR. Control primers corresponding to Translation initiation factor *if-1* (BR0249) were used: IF-1_Fw and IF-1_Rv^65^.

#### Genomic mapB3xFLAG tagging

An amplicon of 719 bp corresponding to the 3′ region of *mapB* ORF was amplified using primers MapB_fw_FLAG and MapB_rev_FLAG, and then subcloned into pQE1-3xFLAG plasmid using the EcoRI restriction enzyme site upstream the 3xFLAG sequence, obtaining the pQE1-3′*mapB*3xFLAG vector. The *mapB* 3′ fragment and the 3xFLAG sequence were cloned into the pK18mobSacB plasmid. The resultant pK18mobSacB3′*mapB*3xFLAG plasmid was transformed into *E*. *coli* S17-1 and then conjugated with the *B*. *suis* wt strain. Selection of double recombinants (Nal^R^, Km^S^, sucrose^R^) was carried out.

### Tolerance to different stresses

Tolerance to acidic pH and different compounds was assayed similarly as previously described^9,10^. Bacteria were incubated either in MM buffer pH 4.5^66^, or with the compound of interest (0.1% DOC, 0.67 mM EDTA, 0.1% Triton X-100, 0.01% SDS), and then serial dilutions were plated in TSA. The CFU were counted, and the percentage of surviving bacteria relative to the control and the wt was calculated (% survival). Oxidative stress resistance by treatment with H_2_O_2_ was evaluated as previously described^67^.

The bactericidal effect of Polymyxin B was tested as follows: 2.5 × 10^5^ CFU of each strain were incubated for 60 min at 37 °C with 0 (control), 10, 50 or 100 units/ml of Polymyxin B in 500 μl of 1 mM HEPES pH 8.0 (Sigma-Aldrich Co.). Tolerance was expressed as the mean percentage of CFU relative to non-treated bacteria.

*B*. *suis* lysozyme tolerance was studied as previously described^68^. Briefly, bacteria cultured in rich medium were harvested at an OD_600_ ≈ 0.6, and suspended in buffer 50 mM Tris-HCl pH 8.0, 2 µg/ml lysozyme at RT. The OD_600_ of the suspension was measured every 10 seconds.

A serum resistance test was performed as previously described^69^. Briefly, bacteria were grown in rich media (TSB) at 37 °C up to early-logarithmic phase (OD_600_ betwen 0.3–0.5). Then the bacteria were washed with PBS-5 mM MgCl_2_ and suspended in the same buffer. A dilution was made with the same buffer in order to obtain a suspension of an OD_600_ of 0.01. Cells were incubated at 37 °C in 50% porcine serum. After 90 min serial dilutions were plated to determine the CFU. The CFU were counted, and the percentage of surviving bacteria relative to the control (time 0) and the wt was calculated (% survival). As control, serum complement was inactivated by incubating the serum at 56 °C for 30 min. The experiment was repeated in triplicate three times with similar results.

### LPS Preparation and analysis

LPS was obtained by two independent protocols similar as previously described: isopropanol precipitation^70^, and hot-phenol extraction^71^. In both cases, after mixing with Laemmli sample buffer, the LPS suspension was analyzed by SDS-PAGE (15%) in Tris-glycine running buffer and visualized by carbohydrate-specific periodate oxidation and silver staining as previously described^72^. Otherwise, after resolving the samples by SDS-PAGE (15%) an immunochemical analysis was performed. Nitrocellulose membranes were blocked with TBS 5% milk powder (w/v) for 2 h at 4 °C with gentle agitation, probed with monoclonal M84 anti-S-LPS antibody^73^ or anti-R-LPS antibody^74^ (1:1,000 and 1:3,000 respectively), and then probed with a goat HRP-conjugated secondary anti-mouse antibody (1:30,000 Santa Cruz). Finally, membranes were developed using ECL Prime (GE Healthcare) and ImageQuant LAS4000 Imager (GE Healthcare Life Sciences).

Crystal Violet (CV) staining of colonies was performed as previously described^75^. Colonies from rough strains are stained darker than smooth strains. Whenever mentioned, *B*. *suis* 1330 and *B*. *ovis* REO 198 strains were included as smooth and rough controls respectively.

### Total lipid extraction and purification

Cultures were grown in Plommet medium^76^ and total lipids were extracted and purified by Bligh and Dyer method^77^. Total lipids were analyzed by GC/MS and TLC.

### Protein preparation and analysis

Total proteins from the different *Brucella* strains were obtained by suspending bacterial pellets in Laemmli sample buffer. *B*. *suis* total membranes were prepared similarly as previously described^78^. Briefly, 100 ml of TSB cultures at an OD_600_≈ 1 were harvested by centrifuging 10 min at 5,000 × *g*, suspended in Protoplasting buffer (15 mM Tris-HCl pH 8.0, 0.45 M sucrose, 8 mM EDTA, 1 mM PMSF and 2 mg/ml lysozyme for the wt strain and 1 mg/ml for the *∆mapB* mutant) and incubated for 20 min on ice. Cells were then centrifuged 10 min at 4,200 × *g* and then suspended in Lysis buffer (50 mM Tris-HCl pH 7.6, 5 mM MgCl_2_, 1 mM PMSF, 10 ug/ml DNaseI, 2ug/ml RNase) and disrupted using Precellys24 (Bertin technologies): 7 cycles of 3 × 30 sec at 6,500 rpm, incubating on ice between each cycle. The homogenate was centrifuged for 2 min at 5,000 × *g* at 4 °C to remove unbroken cells and precellys beads, and the supernatant was then centrifuged at 5,000 × *g* for 30 min and then ultracentrifuged at 100,000 × *g* for 90 min at 4 °C. The pellet was washed with 50 mM Tris HCl pH 8.0 and ultracentrifuged again. Laemmli sample buffer with 8 M urea was used to suspend the pellet. Samples were then heated for 10 min at 95 °C and subjected to SDS-PAGE electrophoresis. When required, total membrane fractions were treated with 5 mg/ml lysozyme for 30 min at 37 °C to digest the remaining peptidoglycan attached.

Protoplast (IM and cytoplasm) were separated from the OM and periplasm as previously described^27^. Briefly, cultures were harvested at exponential growth phase (OD_600_ ≈ 1). 2.5 × 10^10^ bacterial cells were centrifuged 10 min at 3,500 × *g*. The pellets were washed with physiological solution, centrifuged for 10 min at 3,500 × *g* and resuspended in 1 ml of 0.2 M Tris-HCl pH 7.6. Subsequently, 1 ml of 0.2 M Tris-HCl pH 7.6, 1 M sucrose and 0.25% Zwitterion 3–16 solution was added to the cell suspension and incubated for 10 min at RT. The samples were centrifuged for 30 min at 8,000 × *g*, the pellets separated from the supernatants and stored at −80 °C until used.

In each fractioning experiment, controls of the purification procedure were carried out by Western blot using anti-PhyR and anti-Omp10^36^ antibodies as cytoplasmic^65^ and membrane protein controls respectively. All samples were resolved by SDS-PAGE and visualized by Coomassie Brilliant Blue or silver staining. Western blot analysis were performed after transferring the proteins to PVDF membranes (H-bond GE Healthcare), blocked ON with TBS 5% milk powder (w/v) at 4 °C with gentle agitation. Blots were probed with undiluted monoclonal mouse anti-OMPs hybridomes anti-Omp1, Omp2b, Omp10, Omp16, Omp19, Omp25 (A68/04B10/F05) and Omp31 (A59/10F09/G10)^36,79^ or primary mouse anti-FLAG M2 monoclonal antibody (SIGMA) for 1 h at RT, followed by a 1 h incubation at RT with goat HRP-conjugated secondary anti-mouse antibody (1:30,000 Santa Cruz), and then developed using ECL Prime (GE Healthcare) in a ImageQuant LAS4000 Imager (GE Healthcare Life Sciences). Gel band quantification was carried out with ImageJ program.

### OMV purification

Cultures of the different strains were grown up to exponential phase. Next, cells were pelleted down at 5,000 × *g* at 4 °C. In order to remove residual cells, the supernatant was filtered using a 0.22-µm SFCA membrane (Minisart®; Sartorius). To harvest the OMVs, the filtrate was subjected to ultracentrifugation at 100,000 × *g* for 6 h at 4 °C (Beckman Coulter). The supernatant was discarded, the pellet containing the OMVs was washed with sterile PBS, and the ultracentrifugation step was repeated. The final pellet was suspended in PBS.

### Proteomic analysis

Protein digestion and Mass Spectrometry analysis were performed at the Proteomics Core Facility (CEQUIBIEM), at the University of Buenos Aires (UBA-CONICET) as follows: whole membrane proteins were digested with 100 ng Trypsin (Promega V5111) in 25 mM ammonium bicarbonate pH 8.0 ON at 37 °C. Peptides were desalted using C18 zip tips (Merck Millipore) and eluted in 10 ul of H_2_O:ACN:FA 40:60:0.1%. The digests were analysed by nanoLC-MS/MS in a nanoHPLC EASY-nLC 1000 (Thermo Scientific) coupled to a QExactive Mass Spectrometer. A 120 min gradient of H_2_O:ACN at a flow of 33 nl/min was used with a C18 2 mm Easy Spray column × 150 mm. Data Dependent MS2 method was used to fragment the top 12 peaks in each cycle. The raw data from mass spectrometry analysis was processed using the Proteome Discoverer – version 2.1.1.21 – (Thermo Scientific) software for database searching with the SEQUEST search algorithm. The search was performed against the *B*. *suis* 1330 database. Precursor mass tolerance was set to 10 ppm and product ion tolerance to 0.05 Da. Static modification was set to carbamidomethylation of Cys, and dynamic modifications were set to oxidation of Met and N-terminal acetylation. Protein hits were filtered for high confidence peptide matches with a maximum protein and peptide false discovery rate of 1% calculated by employing a reverse database strategy.

In order to perform Label free quantification, samples were analyzed by triplicate. The data obtained for the area for each protein were processed with the Perseus program (Max Planck Institute of Biochemistry, 1.5.5.3 version, available for free) which allows a deeper statistical analysis. For each couple of samples, we plotted -log p-value (-Log Student T-test p-value A_B) on the *y* axis versus Student T-test Difference A_B in *x* axis. Proteins that appear in the volcano plot with a fold change greater than 2 (less than -1 or greater than 1 on the *x* axis of the graph) and a p-value below 0.05 (above 1.3 on the *y* axis of the graph) were considered as differentially expressed.

### Electron microscopy

For TEM analysis, bacterial cultures at an OD_600_ ≈ 2.5 were harvested, washed twice with phosphate buffer and fixed in 2% v/v glutaraldehyde (SIGMA GradeI 25%) in phosphate buffer pH 7.2–7.4 for 2 h at 4 °C. Secondary fixation was performed with 1% osmium tetroxide for 1 h at 4 °C and then the samples were dehydrated in increasing ethanol concentrations and included in an epoxy resin. Ultrathin sections (90 nm) were contrasted with uranyl acetate and lead citrate. The samples were examined in the transmission electron microscope JEM 1200 EX II (JEOL) in the Central Service of Electron Microscopy at the Faculty of Veterinary Sciences, UNLP.

For SEM analysis, bacterial cultures at an OD_600_ ≈ 2.5 were fixed in 2% v/v glutaraldehyde (SIGMA GradeI 25%) for 15 min at RT, and mounted in coverslips pretreated with poly-D-lysine. The samples were dehydrated in increasing ethanol concentrations (from 20 to 100%, 3 times each concentration, for 5 min each time)^80^. After critical point drying and metallization, samples were mounted into the grids with carbon tape and observed at Center of Advance Microscopy (CMA- FCEyN UBA) using a Carl Zeiss NTS SUPRA 40.

### Cell infection assays

Cell infection assays were carried out similarly as previously described^27^. Briefly, J774A.1 murine macrophages cells were seeded in 24-well plates and inoculated with the different *Brucella* strains (MOI 1:100). Intracellular viable bacteria were determined by standard gentamicin-protection assay followed by cell lysis with 0.1% Sodium Deoxycolate (DOC) and plating serial dilutions.

### Virulence in BALB/c mice

Animal procedures and management protocols were approved by the local Institutional Animal Welfare Committee (“Comité de Bienestar Animal”, protocol number 225/2015) according to Animal Welfare Policy (Act 087/02) of the Facultad de Ciencias Veterinarias, Centro de Investigación Veterinaria (CIVETAN).

Six to eight weeks-old female BALB/c mice were purchased from the animal facility at Leloir Institute. They were randomly distributed in experimental groups at least 1 week before being inoculated. The animals were housed in a filter-ventilated containment in the Laboratory Animal Facility of the Facultad de Ciencias Veterinarias, Centro de Investigación Veterinaria (CIVETAN), CONICET-Facultad de Ciencias Veterinarias, Universidad Nacional del Centro de la Provincia de Buenos Aires (U.N.C.P.B.A) receiving water and food *ad libitum*. All experimental protocols were performed by the premise of minimizing the suffering to which animals are exposed and using the minimum number of experimental animals to ensure statistically significant results. BALB/c mice (16 per group) were inoculated by intragastric delivery with *B*. *suis* wt (1.30 × 10^8^ CFU/mouse), Δ*mapB* mutant strain (1.23 × 10^8^ CFU/mouse) or the complemented Δ*mapB* pBBR*mapB* strain (1.29 × 10^8^ CFU/mouse), suspended in 300 µl of 10% sodium bicarbonate by use of a plastic feeding tube introduced through the mouth. Five mice from each group were sacrificed at 7, 15 and 45 days post infection (p.i.), and spleens were removed. Serial dilutions of spleen homogenates were plated in duplicate on TSA and the CFU were counted after 4 days post incubation at 37 °C. The CFU data was normalized by log transformation and evaluated by one-way ANOVA, followed by Tukey’s *posteriori* test (Prism 5.0; GraphPad Software, Inc.). Data is shown as the log_10_ (CFU/spleen). The experiment was repeated twice with similar results.

### Bioinformatic analysis

Domains search was carried out using Pfam^81,82^. Signal peptide sequence and subcellular localization were predicted using SignalP Server^83^ and Gneg-mploc^84^. Alignments were performed with MUSCLE^85^. Transmembrane segments were predicted with TMpred transmembrane prediction^86^, and molecular weight was predicted using Compute pI/Mw from ExPASy^87^. Operon prediction was carried out using MicrobesOnline Operon Predictions^88^. Secondary structure was predicted by Phyre^89^ and Jpred 4^90^.

## Supplementary information

Supplementary information
