# Supplementary material for: MapB, the Brucella suis TamB homologue, is involved in cell envelope biogenesis, cell division and virulence
Source: Sci Rep. 2019 Feb 15;9:2158. doi: 10.1038/s41598-018-37668-3 (PMC6377625; doi:10.1038/s41598-018-37668-3)
Supplement: Supplementary file 1 — Supplementary information [file 41598_2018_37668_MOESM1_ESM.pdf]

MapB, the *Brucella suis* TamB homologue, is involved in cell envelope biogenesis, cell division and virulence

Magalí Graciela Bialer[1‡], Verónica Ruiz-Ranwez[1‡], Gabriela Sycz[1], Silvia Marcela Estein[2], Daniela Marta Russo[1], Silvia Altabe[3], Rodrigo Sieira[1], and Angeles Zorreguieta[*14]

Supplementary information:

1. **Supplementary figures**
   Fig. S1
   Fig. S2
   Fig. S3
   Fig. S4
   Fig. S5

2. **Supplementary tables**
   Table S1
   Table S2
   Table S3
   Table S4
   Table S5

Figure S1

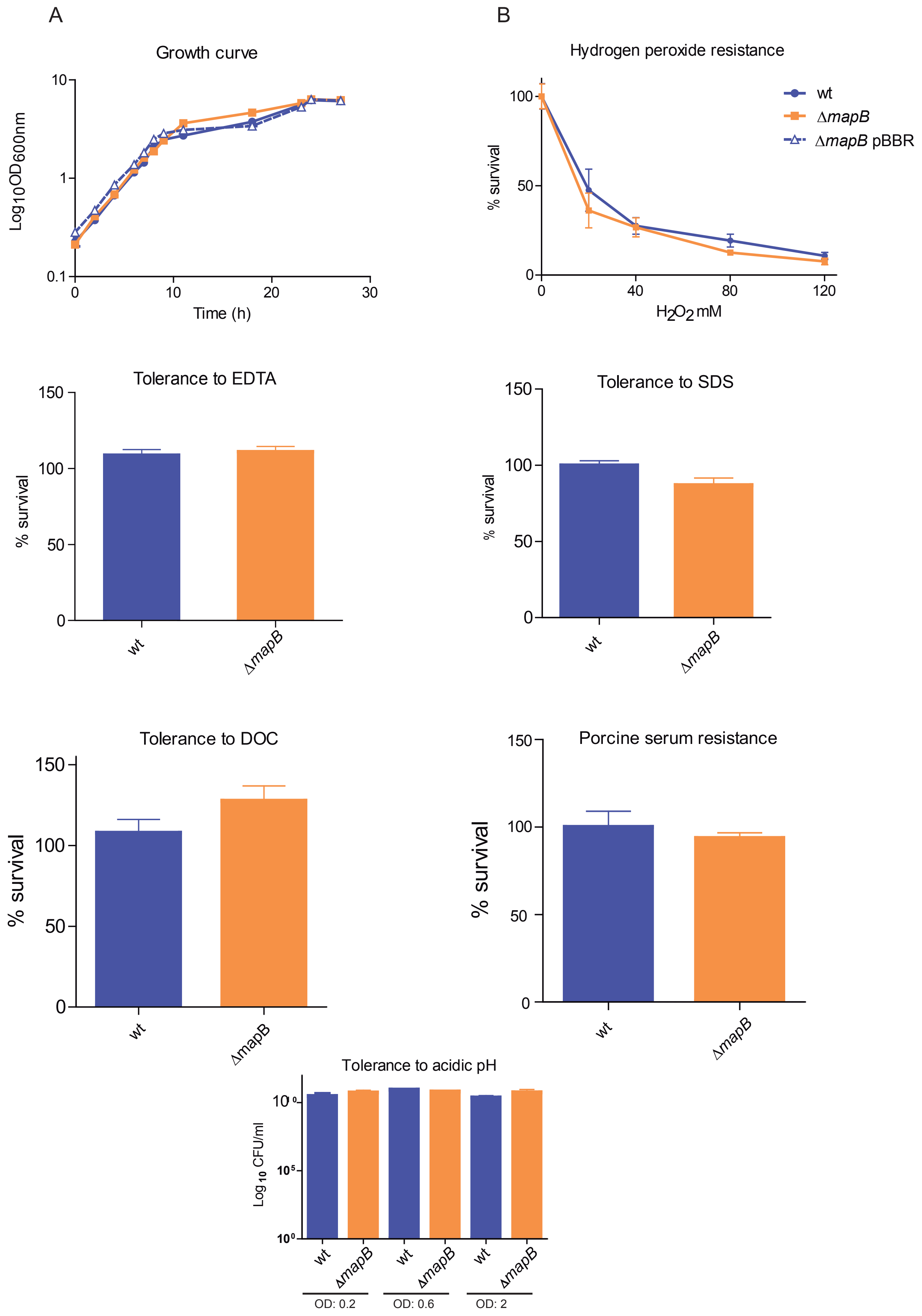
A
Growth curve
Log10OD600nm
10
1
0.1
0
10
20
30
Time (h)
B
Hydrogen peroxide resistance
% survival
100
50
0
0
40
80
120
H2O2 mM
wt
ΔmapB
ΔmapB pBBR
Tolerance to EDTA
% survival
150
100
50
0
wt
ΔmapB
Tolerance to SDS
% survival
150
100
50
0
wt
ΔmapB
Tolerance to DOC
% survival
150
100
50
0
wt
ΔmapB
Porcine serum resistance
% survival
150
100
50
0
wt
ΔmapB
Tolerance to acidic pH
Log10 CFU/ml
10
10⁵
10⁰
wt
ΔmapB
wt
ΔmapB
wt
ΔmapB
OD: 0.2
OD: 0.6
OD: 2

Fig. S1. Growth curve and tolerance to stresses. (A) *B. suis* 1330 (wt), Δ*mapB* and the complemented Δ*mapB* pBBR*mapB* strains were grown at 37°C in a shaker at 200 r.p.m in rich media, and the OD_{600} was registered at different time points. (B) Bacterial strains were incubated with 0, 20, 40, 80 or 120 mM of hydrogen peroxide for 1 h, and CFU were determined. For both strains, the CFU at each treatment were normalized to the CFU obtained at 0 mM of H_2O_2 and the % of survival is shown. (C) *B. suis* M1330 (wt) and the isogenic Δ*mapB* strains were analyzed. Bacteria were incubated with or without 0.1% (C) 0.67mM EDTA (D) 0.01% SDS or (E) or DOC, for 10 min at RT, and CFU were determined. For both strains, the CFU obtained after the incubation with each compound were normalized to the control (CFU obtained without treatment). The % of survival of each strain relative to the wt is shown. Three independent experiments were performed with similar results. Data was analyzed by Student´s T-test and one-way ANOVA with no significantly different results. (F) Porcine serum resistance was performed. The CFU were counted, and the percentage of surviving bacteria relative to the control (time 0) and the wt was calculated (% survival) (G) Tolerance to acidic pH was carried out at OD: 0.2, 0.6, and 2. CFU obtained after the incubation were normalized to the control (CFU obtained without treatment). The % of survival of each strain relative to the wt is shown.

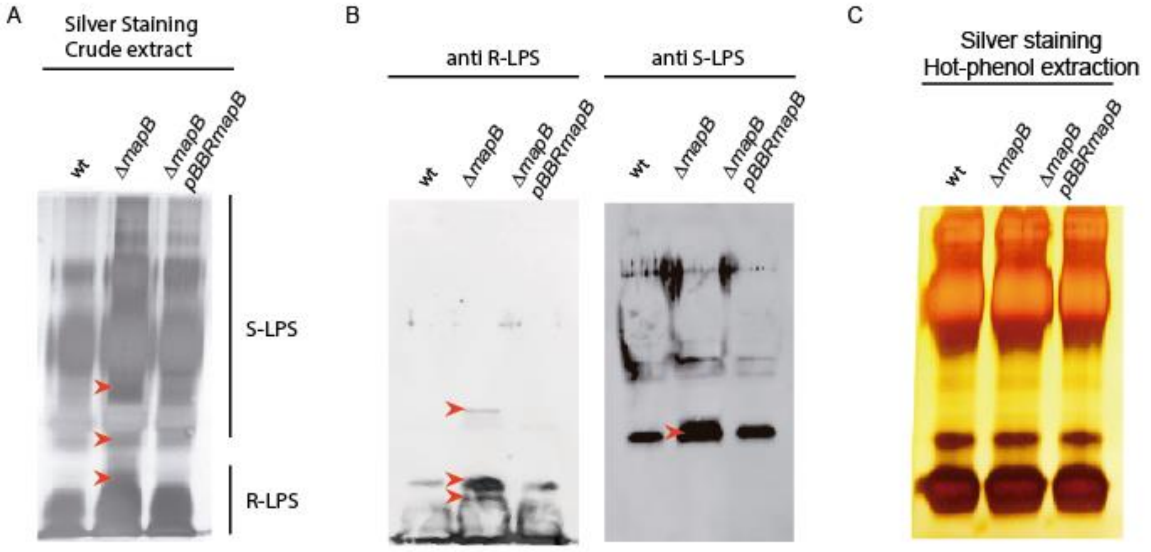

Fig. S2. Global LPS analysis reveals no major changes in the Δ*mapB* mutant polymer. (A) SDS-PAGE and silver staining of crude extracts of LPS from each strain. (B) Western blot analysis performed with anti-R-LPS (left panel) or anti-S-LPS (right panel) antibodies. Red arrows indicate the presence of extra or stronger bands in the Δ*mapB* mutant LPS. (C) SDS-PAGE and silver staining of LPS extracted with hot-phenol protocol from the *B. suis* M1330 (wt), Δ*mapB* and the complemented Δ*mapB* pBBR*mapB* strains.

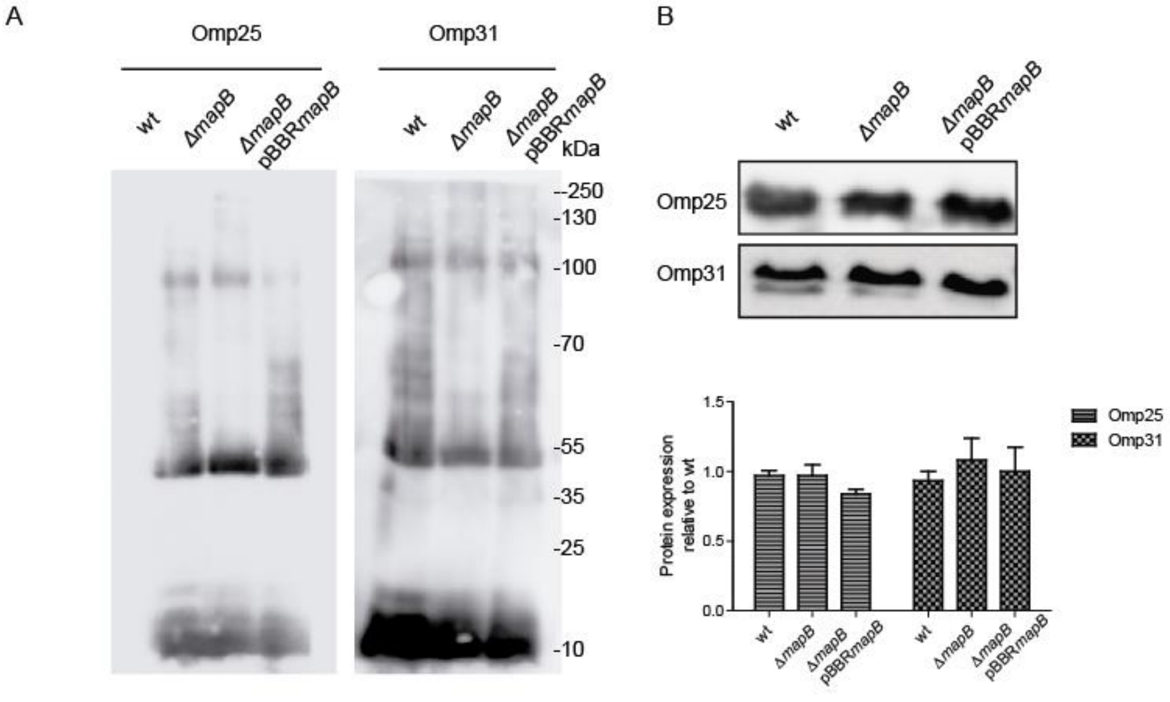

Fig S3. Omp 25/31 Western blot analysis. (A) Western blot of total membranes of *B. suis* 1330 (wt), Δ*mapB* and the complemented Δ*mapB* pBBR*mapB* strains with anti-Omp25 and anti-Omp31 (with no treatment post extraction). (B) Western blot of whole cell lysates of strains showed in (A) with anti-Omp25 and anti-Omp31. Quantification of OMPs abundance from three independent experiments was determined relative to OMP10 as loading control (not shown), and the relative OMP abundance in Δ*mapB* is represented as protein expression relative to the wild-type strain. Data was analyzed by one-way ANOVA followed by a Tukey´s *posteriori* test. *: significantly different from the control (wt), ($p < 0.05$).

Figure S4

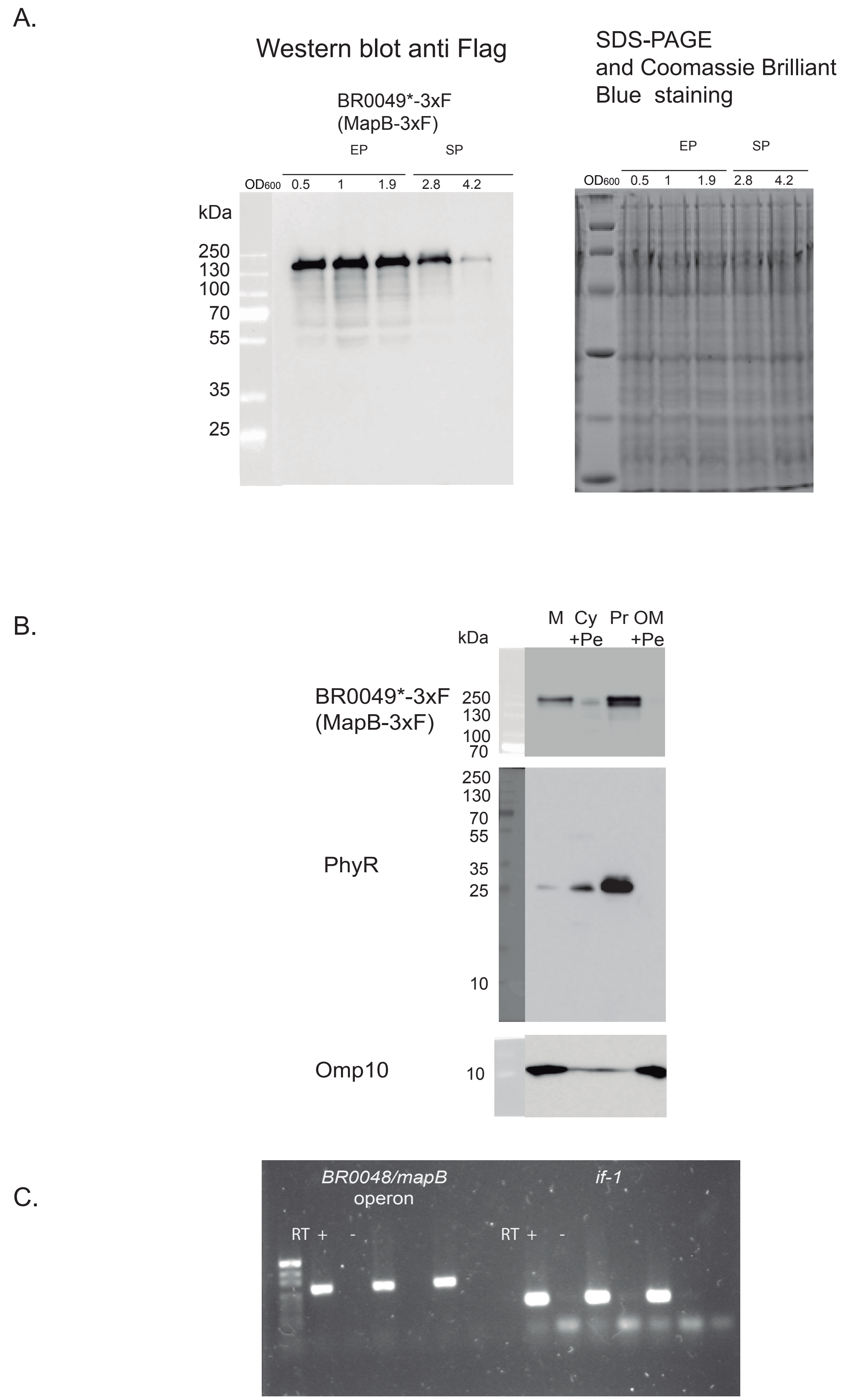

Supplementary Fig. S4. Complete blots and gels from Fig. 1. (A) Full length membrane, blot and gel from Fig.1B. (B) Full length membranes and blots from Fig.1C. MapB-3xF and Omp10 were analyzed on the same blot. Molecular weights of prestained protein standards are shown. (C) Full length agarose gel from Fig. 1D is shown.

Figure S5

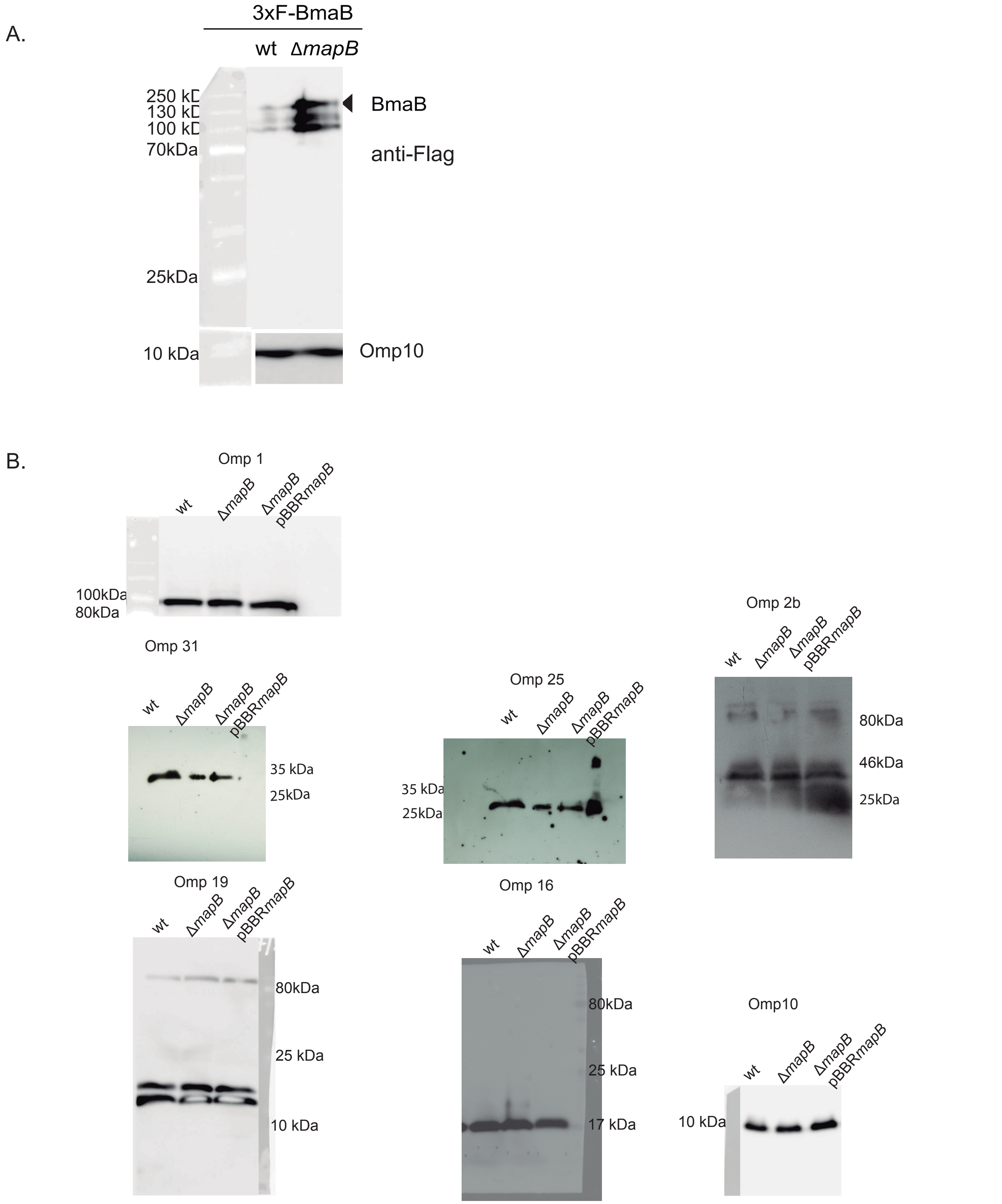

Supplementary Fig. S5. Full length blots from Fig. 3 are shown. Molecular weights of protein standards are shown. (A) Full length blots from Fig. 3A are shown. The 3xF-BmaB protein and the loading control (Omp10) were analyzed on the same blot. (B) Full length blots corresponding to Fig. 3D are shown. According with the target molecular weight the membranes were eventually cut horizontally before blotting.

2. Supplementary Tables

Table S1. Fatty acids analysis

	wt		*ΔmapB*		*ΔmapB pBBRmapB*	
FA	Mean	SD ±	Mean	SD ±	Mean	SD ±
16:01	0.13	0.02	0.09	0.01	0.1	0.01
16:00	2.34	0.17	1.94	0.44	2.09	0.48
17:01	0.11	0.01	0.09	0.02	0.08	0.04
17:00	0.26	0.05	0.18	0.07	0.18	0.09
18:02	0.24	0.05	0.26	0.04	0.3	0.06
18:01	40.86	1.68	26.24	1.49	29.25	1.98
18:00	4.49	0.79	2.44	0.18	2.31	0.12
19:c	50.54	2.76	67.57	1.68	64.78	1.87
20.1	1.18	0.04	1.33	0.11	1.07	0.11
20.0	0.03	0.01	0.03	0.01	0.03	0.01

GC-MS analysis of fatty acids (FA) obtained from *B. suis* 1330 (wt), Δ*mapB* and the complemented Δ*mapB* pBBR*mapB* strains. The results shown correspond to data of three independent experiments.

Table S2. Proteins with other than OM localization significantly diminished

Accession	Gene code	Protein name/Description	*p*-value	Fold change	Localization
A0A0H3G969	BR2114	Uncharacterized protein (DUF 2852 DUF 702 DUF 4140)	0.027	6.5	P
A0A0H3G416	BR1601	Aminotransferase. DegT/DnrJ/EryC1/StrS family protein	0.023	2.75	C
A0A0H3G2C1	BR0564	Uncharacterized protein. L,D-transpeptidase catalytic domain. ErfK/SrfK family protein	0.033	3.9	IM
A0A0H3G767	BRA0535	Oligopeptide ABC transporter, permease protein	0.041	7.3	IM

Proteins with other than OM localization detected by LFQ proteomics that were diminished in *ΔmapB* strain*.* Predicted subcellular localization is indicated: cytoplasm (C), inner membrane (IM) and periplasm (P).

Table S3. OMVs associated proteins

Accession	Gene Code	Protein name/Description	Localization
Wild type			
P0A343	BRA0196	10 kDa chaperonin groS	C
Q8FYR5	BR1251	ATP synthase subunit beta atpD	C
Q8FX87	BRA0195	60 kDa chaperonin groL	C
A0A0H3G431	BR1182	Uncharacterized protein	IM
A0A0H3G988	BR2149	DNA starvation/stationary phase protection protein Dps	C
A0A0H3G433	BR1622	Omp31b	OM
A0A0H3G313	BR0971	Omp25b	OM
ΔmapB			
A0A0H3GC63	BR1284	Omp22	OM
Q45689	BR0701	Omp25	OM
A0A0H3G4S3	BR119	Omp25c	OM
P0A3U5	BRA0423	Omp31	OM
Q8FX87	BRA0195	60 kDa chaperonin,groL	C
A0A0H3G313	BR0971	Omp25b	OM
A0A0H3G433	BR1622	Omp31b	OM
Q8FXX2	BR2125	Chaperone protein DnaK	C
P0DI95	BR0639	Omp2b	OM
A0A0H3GA04	BRA0265	Sugar ABC transporter, periplasmic sugar-binding protein	P
Q8FYR5	BR1799	ATP synthase subunit beta, atpD	IM
A0A0H3GAL2	BRA0538	Oligopeptide ABC transporter, periplasmic oligopeptide-binding protein	P
P64025	BR1251	Elongation factor Tu tufA	C
A0A0H3G7C2	BR1205	Putative lipoprotein	IM/ extracell
A0A0H3G9H5	BR0093	Aconitate hydratase acnA	IM
A0A0H3G7V2	BRA0858	Ribose ABC transporter, periplasmic D-ribose-binding protein rbsB-2	P
Q8FWU0	BRA0355	Catalase katA	P
A0A0H3GFM9	BRA0756	Iron compound ABC transporter, periplasmic iron compound-binding protein	P
Q8FYR3	BR1801	ATP synthase subunit alpha atpA	IM
A0A0H3GAT8	BRA0633	Uncharacterized protein	C
A0A0H3G3E6	BR1204	OmpA/MotB	OM
A0A0H3G3Y9	BR1562	Uncharacterized protein	OM
A0A0H3G4E4	BR1378	Aminotransferase, class I	C
A0A0H3G415	BR1154	BamA	OM
A0A0H3G6H2	BRA0537	Oligopeptide ABC transporter, periplasmic oligopeptide-binding protein	P

Q8FYT2	BR1778	4-hydroxy-3-methylbut-2-en-1-yl diphosphate synthase (flavodoxin)	IM/C
Q8FZ07	BR1697	Tol-Pal system protein TolB	P
Q8G075	BR1236	Elongation factor fusA	C
Q8FUM7	BRA1193	Leu/Ile/Val-binding protein homolog 5	P
Q8G069	BR1243	DNA-directed RNA polymerase subunit beta rpoB	IM/C
A0A0H3G6N4	BRA0632	Amino acid ABC transporter, periplasmic amino acid-binding protein	P
A0A0H3G7W5	BR1199	Isocitrate dehydrogenase	C
A0A0H3G8F4	BR1728	Glyceraldehyde-3-phosphate dehydrogenase	C
Q7CEG6	BR1864	Chaperone protein ClpB	C
Q8FW10	BRA0655	sn-glycerol-3-phosphate-binding periplasmic protein UgpB	P
A0A0H3G7R0	BRA0804	Nickel ABC transporter, nickel-binding protein	P
Q8G094	BR1209	DNA-directed RNA polymerase subunit alpha rpoA	IM
Q8FW84	BRA0576	Putative binding protein	P

OMVs associated proteins detected by nano LC-MS/MS coupled to a QExactive Mass Spectrometer produced by *B. suis* 1330 (wt) and Δ*mapB* strains. Proteins were sorted in order of decreasing score. Predicted subcellular localization is indicated: cytoplasm (C), inner membrane (IM), outer membrane (OM) and periplasm (P).

Table S4. List of strains and plasmids used in this study.

Strain or plasmid	Description and/or relevant phenotype	Reference
Strains		
E. coli		
DH5α	*LacZ*ΔM15 *recA1 hsdR17 supE44 thi-1 gyrA relA1*	Invitrogen
S17-1	Movilization host: cells enable movilizaton of pBBR plasmids from *E. coli* S17-1 to *Brucella* strains. *recA, thi, pro,* $hsdR^-M^-$ RP4 : 2-Tc:Mu: Km Tn7 λpir.	*Escherichia coli* (ATCC® 47055™)
Brucella suis		
wt M1330	Wild-type, smooth, virulent, Nal^R	Laboratory stock
Δ*mapB*	M1330 strain in which the *mapB* gene (BR0049) has been completely deleted	This work
Δ*mapB pBBRmapB*	Δ*mapB* mutant strain complemented with the pBBR1MCS-1-*mapB* vector, which expresses *mapB* under the control of its own promoter	This work
mapB3xflag	M1330 strain in which the *mapB* gene (BR0049) has been tagged in the 3' end with a 3xflag sequence	This work
wt_*pBBR3xflagbmaB*	M1330 strain with the pBBR*3xflagbmaB* vector, which expresses *bmaB* under the control of *lacZ* promoter and *bmaB* has been tagged with a *3xflag* sequence in the 5'end	This work
Δ*mapB_pBBR3xflagbmaB*	Δ*mapB* mutant strain with the pBBR*3xflagbmaB* vector, which expresses *bmaB* under the control of *lacZ* promoter and *bmaB* has been tagged with a *3xflag* sequence in the 5'end	This work
Plasmids		
pk18mobsacB	Km^R. Mobilizable and suicide vector in *Brucella*, containing the counterselection marker *sacB*	(1)
pk18mobsacB_Δ*mapB*	Km^R. Derivative of pk18mobsacB containing the flanking regions of *mapB* for Δ*mapB* mutant strain construction	This work
pQE1-*3xflag*	Amp^R. Derivative of pQE1 vector containing the *3xflag* sequence (5'GACTACAAAGACCATGACGGTGATTATAAAGATCATGACATCGATTACAAGGATGACGATGACAAG 3').	A kind gift of Dr. Juan Manuel Spera, UNSAM.
pQE1-3'*mapB3xflag*	Amp^R. Derivative of pQE1-*3xflag* vector containing the *3xflag* sequence downstream the 3' end of *mapB*	

pk18mobsacB_3'*mapB3xflag*	Km^R. Derivative of pk18mobsacB containing the 3' end of *mapB* gene and the *3xflag* sequence, used for *mapB3xflag* strain construction	This work
pBBR1MCS-1	Cm^R. pBBR1MCS-1 Broad-host-range cloning vector of mid-number- copy	(2)
pBBR*mapB*	Cm^R. pBBR1MCS-1-*mapB* vector, which expresses *mapB* under the control of its own promoter	This work
pBBR*3xflagbmaB*	Cm^R. pBBR1MCS-1-*3xflagbmaB* vector, which expresses *3xflagbmaB* under the control of *lacZ* promoter, *bmaB* has been tagged with a *3xflag* sequence in the 5'end	This work

Table S5. List of primers used in this study.

Oligonucleotides name	5' to 3' sequences*	Restriction Site
ΔmapB_F1	GAATTCTCTCCGAGCAGATGCGCGTCG	EcoRI
ΔmapB_R1	GGATCCGCCTGCGGCTGGTCATCAGC	BamHI
ΔmapB_F2	GGATCCTATGAGCGGCCATTACGGC	BamHI
ΔmapB_R2	TCTAGATGATCCTTCAGGCTGACGACA	XbaI
FComp	CTCGAGGCAATATCCAGCCCTTCTATGAAT	XhoI
RComp	ACTAGTAATTAAGGAATCGGGCCAAAGCAGGC	SpeI
FRTmapB	ATGAAGGGCGATGAAATCAC	-----
RRTmapB	GGCTTGAACGTATTGGCAAG	-----
FRT48	GGCTTCGGCGCTGAATATTC	-----
RRT48	TATCCTGCGAGCCGCCAATG	-----
FRT50	GAACCATTGTCGTCAGCCTG	-----
RRT50	TATTCCACATTGGCGCGGCC	-----
RtOpF	GCTCCGGCGATCCGAACTAT	-----
RtOpR	TGGAGATACGGCGGTTGGGT	-----
IF_Fw	TGTTACGGAACTGCTGCCCAAT	-----
IF_Rv	CGGCCCTTGGTCAGGTCATAA	-----
MapB_fw_FLAG	GAATTCACTGTTCCGCGTCTTGATG	EcoRI
MapB_rev_FLAG	GAATTCATAGTCCTTTTCATAGAAGATG	EcoRI

*Underlined nucleotides correspond to restriction enzymes recognition sites.

References

1. Schafer A*, et al.* (1994) Small mobilizable multi-purpose cloning vectors derived from the Escherichia coli plasmids pK18 and pK19: selection of defined deletions in the chromosome of Corynebacterium glutamicum. *Gene* 145(1):69-73.
2. Kovach ME, Phillips RW, Elzer PH, Roop RM, 2nd, & Peterson KM (1994) pBBR1MCS: a broad-host-range cloning vector. *Biotechniques* 16(5):800-802.
